# Making and breaking of boron bridges in the pectic domain rhamnogalacturonan‐II at apoplastic pH *in vivo* and *in vitro*

**DOI:** 10.1111/tpj.16112

**Published:** 2023-02-08

**Authors:** Rifat Ara Begum, David J. Messenger, Stephen C. Fry

**Affiliations:** ^1^ The Edinburgh Cell Wall Group Institute of Molecular Plant Sciences, The University of Edinburgh Daniel Rutherford Building, The King's Buildings, Max Born Crescent Edinburgh EH9 3BF UK; ^2^ Present address: Department of Biochemistry and Molecular Biology, Faculty of Biological Sciences University of Dhaka Curzon Hall Dhaka 1000 Bangladesh; ^3^ Present address: Unilever U.K. Central Resources Limited Colworth Science Park Sharnbrook MK44 1LQ UK

**Keywords:** apiose, apoplastic pH, borate diesters, rhamnogalacturonan‐II, pectin, cell‐wall polysaccharides

## Abstract

Cross‐linking of the cell‐wall pectin domain rhamnogalacturonan‐II (RG‐II) via boron bridges between apiose residues is essential for normal plant growth and development, but little is known about its mechanism or reversibility. We characterized the making and breaking of boron bridges *in vivo* and *in vitro* at ‘apoplastic’ pH. RG‐II (13–26 μm) was incubated in living *Rosa* cell cultures and cell‐free media with and without 1.2 mm H_3_BO_3_ and cationic chaperones (Ca^2+^, Pb^2+^, polyhistidine, or arabinogalactan‐protein oligopeptides). The cross‐linking status of RG‐II was monitored electrophoretically. Dimeric RG‐II was stable at pH 2.0–7.0 *in vivo* and *in vitro*. *In‐vitro* dimerization required a ‘catalytic’ cation at all pHs tested (1.75–7.0); thus, merely neutralizing the negative charge of RG‐II (at pH 1.75) does not enable boron bridging. Pb^2+^ (20–2500 μm) was highly effective at pH 1.75–4.0, but not 4.75–7.0. Cationic peptides were effective at approximately 1–30 μm; higher concentrations caused less dimerization, probably because two RG‐IIs then rarely bonded to the same peptide molecule. Peptides were ineffective at pH 1.75, their pH optimum being 2.5–4.75. d‐Apiose (>40 mm) blocked RG‐II dimerization *in vitro*, but did not cleave existing boron bridges. *Rosa* cells did not take up d‐[U‐^14^C]apiose; therefore, exogenous apiose would block only *apoplastic* RG‐II dimerization *in vivo*. In conclusion, apoplastic pH neither broke boron bridges nor prevented their formation. Thus boron‐starved cells cannot salvage boron from RG‐II, and ‘acid growth’ is not achieved by pH‐dependent monomerization of RG‐II. Divalent metals and cationic peptides catalyse RG‐II dimerization via co‐ordinate and ionic bonding respectively (possible and impossible, respectively, at pH 1.75). Exogenous apiose may be useful to distinguish intra‐ and extra‐protoplasmic dimerization.

## Significance Statement

This study demonstrates the nature of the interaction between the cell‐wall pectic domain rhamnogalacturonan‐II (RG‐II) and cationic chaperones necessary for efficient RG‐II dimerization via borate diester bonds at acidic pH, resembling the acid growth conditions in the cell wall. Boron bridging is irreversible at apoplastic pH, so boron‐starved cells cannot salvage boron from RG‐II, and wall loosening in ‘acid growth’ is not achieved by pH‐dependent modification of RG‐II cross‐linking.

## INTRODUCTION

### Background to rhamnogalacturonan‐II

Rhamnogalacturonan‐II (RG‐II) is a polysaccharide domain of pectin found in all land‐plant primary cell walls (Darvill et al., 1978; Matoh et al., 1996; Matsunaga et al., 2004; O'Neill et al., 1990, 2004). It is a small (approximately 5 kDa) but highly complex domain comprising an oligogalacturonide backbone (approximately seven to 10 residues) bearing six side‐chains, most of which are themselves also acidic (Darvill et al., 1978; Matsunaga et al., 2004; Ndeh et al., 2017; Pabst et al., 2013; Séveno et al., 2009). It forms a covalently contiguous structure with homogalacturonan and possibly RG‐I (Harholt et al., 2010; Whitcombe et al., 1995). A preliminary picture of the three‐dimensional structure of RG‐II has been offered (Jarvis, 2002).

The dimerization of RG‐II via tetrahedral borate diester bonds (boron bridges) (Ishii et al., 1999; Ishii & Matsunaga, 1996; Hu & Brown, 1994; Kobayashi et al., 1996; Kobayashi et al., 1997; Loomis & Durst, 1992; Matoh et al., 1993; O'Neill et al., 1996) underlies its key biological roles, including wall assembly, cell expansion, and wall porosity (Brown & Hu, 1996; Fleischer et al., 1999). For example, boron‐deficient cell walls have a larger pore size, and continue growing beyond the normal maximum size until the cells burst (Fleischer et al., 1998). The boron bridges form between the apiose residue of side‐chain A of one RG‐II molecule and the equivalent site on a neighbouring RG‐II (Ishii et al., 1999; Shimokawa et al., 1999).

The absence of boron bridges, due either to boron mineral deficiency or to genetic modification of side‐chain A, leads to defects in cell expansion, wall permeability and frost tolerance, and to short thick stems and roots, dying growing points, and ruptured epidermal surfaces (Iwai et al., 2002; Lehto et al., 2010; Panter et al., 2019; Reuhs et al., 2004; Warington, 1923; Wimmer & Eichert, 2013).

### Stability of boron‐bridged RG‐II at pH 2–7 *in vivo* and *in vitro*

RG‐II is synthesized in the Golgi system, then secreted into the apoplast. Both these subcellular sites are relatively acidic. Typically, the pH decreases gradually from Golgi cisternae to the *trans*‐Golgi network, from 6.5 to 5.5 (Paroutis et al., 2004; Wu et al., 2000). The apoplast often has pH 5.1–6.5, which can be lowered to approximately 4.5 during auxin‐induced ‘acid growth’ (Felle, 1998; Rayle & Cleland, 1992). Very low apoplastic pH is relevant in some naturally acidic waters (e.g. pH as low as 2.3; Rossini Oliva et al., 2009, 2018). Therefore, the ability of RG‐II to remain stably boron‐bridged, or to undergo *de‐novo* boron bridging, in acidic environments is biologically relevant.

Interest in the possible breakage of boron bridges under physiological conditions is twofold: (i) theoretically, boron‐starved tissues might cleave some pre‐formed boron bridges in the apoplast and thereby salvage traces of boron to be utilized in future growth and development; (ii) cleaving boron bridges in the apoplast at low pH might partially disrupt wall architecture and increase wall extensibility.

It has been claimed that there is an RG‐II dimer ↔ monomer interconversion, and that at pH 3–4 (which could be encountered in the apoplast) the equilibrium lies in favour of the monomer, whereas the dimer is stable at higher pH (O'Neill et al., 1996). Our preliminary work did not support the occurrence of such interconversion. We have therefore now explored in detail the acid stability of boron bridges. Dimeric RG‐II is well established to be monomerized by extreme acidity (pH approximately 1, e.g. 0.1 m HCl), often used experimentally to generate pure monomer. However, the ability of physiologically relevant (apoplastic) acidic pH values to break boron bridges *in vivo* required testing, and we have now explored this both *in vivo* and in model experiments *in vitro*.

Furthermore, in view of the very high susceptibility of apiosyl linkages to acid hydrolysis (Longland et al., 1989; Watson & Orenstein, 1975), we have tested whether the polysaccharide structure of RG‐II remains intact under physiologically relevant acidity.

### Does dimerization of anionic RG‐II require active attraction or just absence of repulsion?

RG‐II is highly anionic, the monomer having a net charge of approximately −15 at near‐neutral pH. Judged from the mobility towards the anode during paper electrophoresis at pH 2.0 (figure 4a of Voxeur & Fry, 2014), the net charge of monomeric RG‐II at pH 2 can be estimated at −0.8 by application of Offord's rules (Fry, 2020; Offord, 1966); therefore, the *p*K_a_ of monomeric RG‐II was taken as approximately 3.23.

Because of their anionic nature, two RG‐II monomer molecules will electrostatically repel one another at most physiological pH values, which would hinder their dimerization via boron bridging. This will apply particularly to the specific apiose residue (in side‐chain A) involved in boron bridging, which is closely flanked by a negatively charged homogalacturonan‐like region (the backbone of RG‐II, most of whose GalA residues are not methylesterified and are therefore anionic; Ndeh et al., 2017; O'Neill et al., 2020) plus a rhamnose residue that carries two additional galacturonate residues [one of which can be methyl‐etherified (O'Neill et al., 2020), which, however, does not prevent it being anionic].

Thus, simply mixing RG‐II with boric acid results in little dimerization (Begum & Fry, 2022; Chormova et al., 2014; Sanhueza et al., 2022). Dimerization of RG‐II in the presence of boric acid can be catalysed by certain cationic ‘chaperones’ such as divalent metal ions (Ishii et al., 1999; O'Neill et al., 1996) and positively charged peptides (Chormova & Fry, 2016; Sanhueza et al., 2022), which are presumed to overcome the electrostatic repulsion between neighbouring RG‐II molecules. Although it had previously been reported (O'Neill et al., 1996) that boric acid can dimerize RG‐II up to 63% within 72 h at pH 3.3 *in vitro* with no chaperones added, this non‐catalysed dimerization was not found appreciably in other studies (Chormova & Fry, 2016; Sanhueza et al., 2022).

For RG‐II dimerization to occur, it is necessary for the electrostatic repulsion to be overcome – but is this *sufficient*? To address this question *in vitro*, we have tested whether boron bridging occurs when the pH of the medium is decreased to a value at which carboxylic acids carry almost no net charge but which is not so extremely acidic as to cleave boron bridges.

If nullifying the negative charge of RG‐II is not sufficient to initiate boron bridging, then it would be concluded that some additional agent must be present to bring together the two RG‐II molecules actively. To explore this concept, we have also tested the ability of six different cationic ‘chaperones’ to catalyse the dimerization of RG‐II at a range of acidic (potentially apoplastic) pH values. The tested chaperones include the metal ions Pb^2+^ and Ca^2+^ (Ishii et al., 1999; O'Neill et al., 1996) and cationic peptides (Chormova & Fry, 2016; Sanhueza et al., 2022).

### Mode of action of divalent metal cations in catalysing RG‐II dimerization: few co‐ordinate bonds versus many ionic bonds

The sequence of reactions leading to boron bridging of RG‐II is currently unclear. Bharadwaj et al. (2020) modelled the boron bridging of a neutral apiose compound, 1,3′‐dimethoxyapiose, on the assumption that it resembles the apiose of RG‐II's side‐chain A. However, this model compound has not been proven to dimerize stably via boron, as seen in RG‐II. 1,3′‐Dimethoxyapiose is not located in a molecular environment rich in negatively charged carboxylic acids, whereas the apiose of side‐chain A of RG‐II has several ionizable galacturonates within two residues. Bharadwaj et al. (2020) suggested that the limiting step for the theoretical boron bridging of 1,3′‐dimethoxyapiose is the first step (reaction of one sugar molecule with one boric acid molecule to form a non‐ionized boric ester), though whether the same applies to RG‐II is currently unknown. Further evidence is needed to elucidate the cross‐linking mechanism of RG‐II.

Previous work has shown that certain divalent metal cations, particularly those with an ionic diameter >0.22 nm (e.g. Pb^2+^, Sr^2+^, and Ba^2+^) serve as excellent chaperones, promoting RG‐II dimerization (Ishii et al., 1999; Jarvis, 2002; Kobayashi et al., 1999; Matoh & Kobayashi, 1998; O'Neill et al., 1996). For example, 0.5 mm Pb^2+^ catalysed up to 98% dimerization of equimolar RG‐II at pH 2.0–4.5, and 0.5 mm Sr^2+^ gave up to 78% dimerization at pH 3.6–4.0 (O'Neill et al., 1996). Lower concentrations of Pb^2+^ (e.g. 0.1 mm, with 0.5 mm RG‐II) were less efficient (Ishii et al., 1999).

The more biologically relevant divalent cation, Ca^2+^, is effective only at much higher concentrations, e.g. 50 mm (Ishii et al., 1999). To date, experiments have tended to use divalent metal cations approximately equimolar to (or exceeding) the RG‐II. Thus, the metal ions could either cancel the negative charges on the RG‐II molecules based on ionic interactions, or a single divalent metal cation could bridge two RG‐II molecules by co‐ordinate bonding, without appreciably decreasing their negative charge. To distinguish these options, we have now tested Pb^2+^ ions at very low concentrations, even less than the RG‐II concentration.

### Mode of action of cationic peptides in causing RG‐II dimerization: abolishing repulsion versus active juxtaposition

Other work has shown that, besides metal cations, polycationic proteins and arabinogalactan‐protein (AGP)‐related oligopeptides also serve as excellent chaperones, promoting RG‐II dimerization via boron bridges. Proteins able to act in this way include artificial polyhistidine (PH), and the glycoproteins AGP31 and extensin (Chormova & Fry, 2016; Sanhueza et al., 2022).

As these are *poly*cations, one molecule could neutralize most or all of the negative charges on a single RG‐II molecule (depending on pH). However, although this would be expected to prevent the mutual repulsion of RG‐II molecules, it would not of itself actively bring together two RG‐II molecules. To explore the possible need for the latter process, we have tested the effect of increasing the concentration of polycations to the point where it becomes improbable that two RG‐II molecules would become seated on the same polycation molecule and thereby actively brought together.

The three oligopeptide chaperones tested in the present paper are remarkably basic oligopeptides from Arabidopsis AGP sequences, referred to here as AGP17p, AGP18p, and AGP19p1 (Sanhueza et al., 2022). For example, AGP19p1 is KHKRKHKHKRHHH, all 13 residues of which are basic. The AGPs that contain these sequences are of interest in studies of pectin because they are located in the two proposed subcellular sites of RG‐II dimerization (Begum & Fry, 2022) – transiently in the Golgi system and permanently in the apoplast. Furthermore, they play roles in plant growth and development as demonstrated by the abnormal morphology of *agp17*, *agp18*, and *agp19* mutants (Sun et al., 2005; Yang et al., 2007, 2011).

We have suggested (Sanhueza et al., 2022) that these AGPs, possessing highly cationic domains, are RG‐II borate diesterase enzymes and represent the first‐reported boron‐acting enzyme activity, catalysing a reaction that covalently makes and breaks borate esters:

$$2\;\mathrm{RG}‐\mathrm{II}‐H_{2}+B{{\left(\mathrm{OH}\right)}_{3}}^{\leftarrow }\to \mathrm{RG}‐\mathrm{II}–B^{–}‐\mathrm{RG}‐\mathrm{II}+3\;H_{2}O+H^{+},$$

in which the equilibrium at physiological pH values lies far to the right.

Although the *in‐vitro* dimerization of RG‐II has been demonstrated with various chaperones (Chormova & Fry, 2016; Ishii et al., 1999; Kobayashi et al., 1999; O'Neill et al., 1996; Pellerin et al., 1996; Sanhueza et al., 2022), the pH dependence of this process had not been methodically explored. The pH is crucial as it determines the ionization both of RG‐II (fully anionic at neutral pH, almost non‐ionized at very low pH) and of basic peptides (fully cationic at low pH, the histidine residues becoming progressively less fully ionized as the pH is raised to 7).

### Inhibitors of apoplastic RG‐II dimerization

For studying the subcellular site(s) of RG‐II dimerization, a valuable tool would be a membrane‐impermeant agent that blocks boron bridging. In the present work we have therefore also explored the effect of monosaccharides possessing a furanose ring with a *cis*‐diol group that can bind boric acid (Weigel, 1963) and potentially interrupt the attachment of boron to RG‐II. We show that apiose fulfils criteria making it useful for this purpose.

## RESULTS

### Boron bridges are stable in living rose cell cultures

We investigated whether boron‐bridged RG‐II domains can be monomerized *in vivo*. Two factors were selected that might potentially promote monomerization, i.e. (i) boron deficiency, and (ii) a low apoplastic pH, such as is encountered during growth in acidic waters or during auxin‐induced ‘acid growth’.

**Figure 1:**
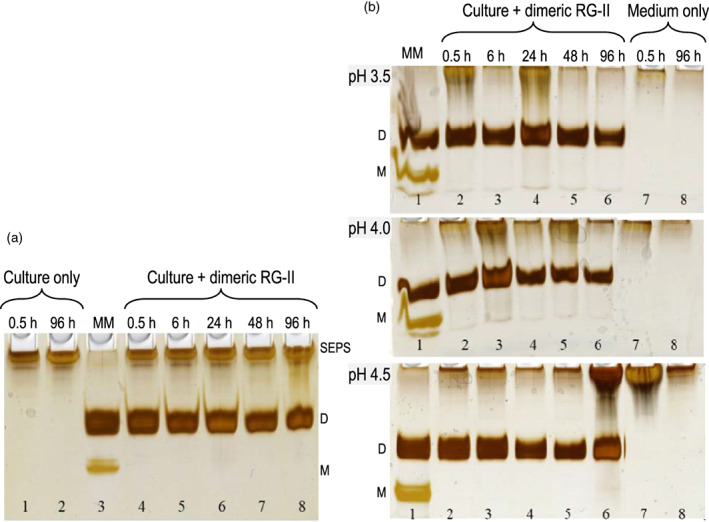
Exogenous dimeric rhamnogalacturonan‐II (RG‐II) does not monomerize in the media of boron‐starved *Rosa* cultures. (a) Unbuffered 3‐day‐old zero‐boron *Rosa* mini‐cultures were axenically fed 13 μm dimeric RG‐II, and at intervals (0.5–96 h) 8 μl of the culture medium was analysed by polyacrylamide gel electrophoresis (lanes 4–8). Lanes 1 and 2 show control cell‐free spent medium without added RG‐II, collected at 0.5 or 96 h. Lane 3 shows a marker mixture (MM) containing RG‐II dimer and monomer. D, dimer; M, monomer; SEPS, soluble extracellular polysaccharides (not characterized here). (b) The experiment was as in (a), with 13 μm dimeric RG‐II, but the cultures (lanes 2–6) were buffered with 25 mm succinate (Na^+^), pH 3.5, 4.0, or 4.5. Lanes 7 and 8 are control cultures without added RG‐II. Lane 1 contains a marker mixture of RG‐II monomer and dimer. pH of the culture medium remained almost constant at the buffered value for the full 96 h of incubation, and initial and final pH values were: 3.50, 3.53; 4.00, 4.03; and 4.50, 4.53. [Two lanes between 6 and 7, not relevant to this experiment, have been deleted from the image.]

- Boron deficiency: dimeric RG‐II was added to zero‐boron *Rosa* mini‐cultures, which were then incubated under standard conditions (pH of medium 5.2) for 0.5–96 h. About 70% of the cells remained viable throughout the experiment (Figure S1). No loss of the soluble extracellular RG‐II dimers was detected, and no soluble RG‐II monomers appeared (Figure 1a). Control cultures with no added RG‐II revealed no soluble extracellular free RG‐II. This confirmed that, although unlikely, there was indeed no free 5‐ or 10‐kDa RG‐II (the only kind that might have interfered with our observations) secreted into the medium. The findings indicate that these boron‐starved cells did not abstract boron from existing apoplastic RG‐II dimers (Figure 1a).
- Low apoplastic pH: dimeric RG‐II was again added to zero‐boron *Rosa* mini‐cultures as above, but the medium was buffered at pH 3.5, 4.0, or 4.5. After 96 h, the cell viability was 60–70% in cultures both at pH 4.0 and at 4.5 (Figure S2). The pH of the medium remained almost constant at the buffered value for the full 96 h of incubation, showing that the succinate buffer was effective. The exogenous dimeric RG‐II did not yield monomers, regardless of the pH of the medium (Figure 1b). As before, no free RG‐II was released into the medium of control cultures.

Thus, extracellular RG‐II dimers are stable *in vivo*, even at physiologically low pH values and in the absence of free boric acid.

### Existing boron bridges are stable *in vitro* at pH values as low as 2

**Figure 2:**
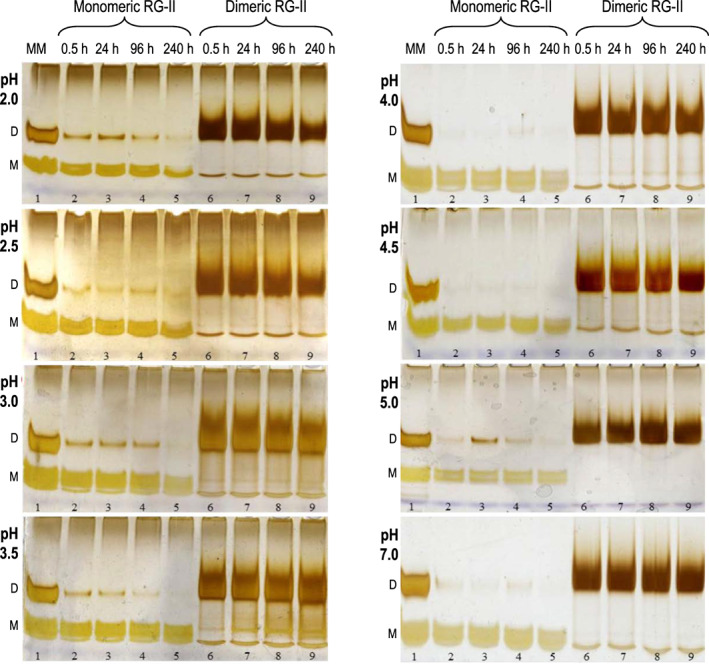
Low pH values *in vitro* cause partial hydrolysis of rhamnogalacturonan‐II (RG‐II) but not cleavage of boron bridges. The experiment was as in Figure 1 but in the absence of cells. Purified RG‐II (26 μm monomer or 13 μm dimer) was incubated in 25 mm pyridine buffers (formate pH 2.0–3.0, or acetate pH 3.5–7.0) for 0.5–240 h, as indicated. Each sample loading (lanes 2–9) contained 4.6 μg of RG‐II and/or its degradation products. Lane 1: marker mixture (MM). D, dimer; M, monomer.

The ‘apoplastic’ pH values (3.5–4.5) studied above *in vivo* did not break boron bridges in the presence of cells. To explore the acid stability of these bridges further, as well as of the polysaccharide itself, we incubated monomeric and dimeric RG‐II at a wider range of pH values, and for longer durations, *in vitro* (Figure 2). The formation of small amounts of dimer from initially pure monomeric RG‐II in some samples (Figure 2) indicates trace boron contamination, and the ability of RG‐II to undergo slight dimerization in the absence of chaperones, although this will not affect the following conclusions.

A slight loss of both monomeric and dimeric RG‐II was noted after 240 h incubation at very low pH values (particularly pH 2.0; Figure 2). The partial loss of dimer was not accompanied by monomer accumulation. Thus, the losses were probably due to partial acid hydrolysis of the polysaccharide's primary structure rather than cleavage of boron bridges, indicating that the boron bridges were stable. This work showed that under the physiological conditions often reported during ‘acid growth’ (e.g. apoplastic pH approximately 4.5; Rayle & Cleland, 1992), dimeric RG‐II is neither monomerized nor hydrolysed.

Although physiologically relevant acidic conditions did not break existing boron bridges in RG‐II dimers, it was considered possible that low pH might inhibit the formation of new boron bridges. This was therefore tested next.

### RG‐II dimerization *in vitro* requires a chaperone, even in the absence of electrostatic repulsion

**Figure 3:**
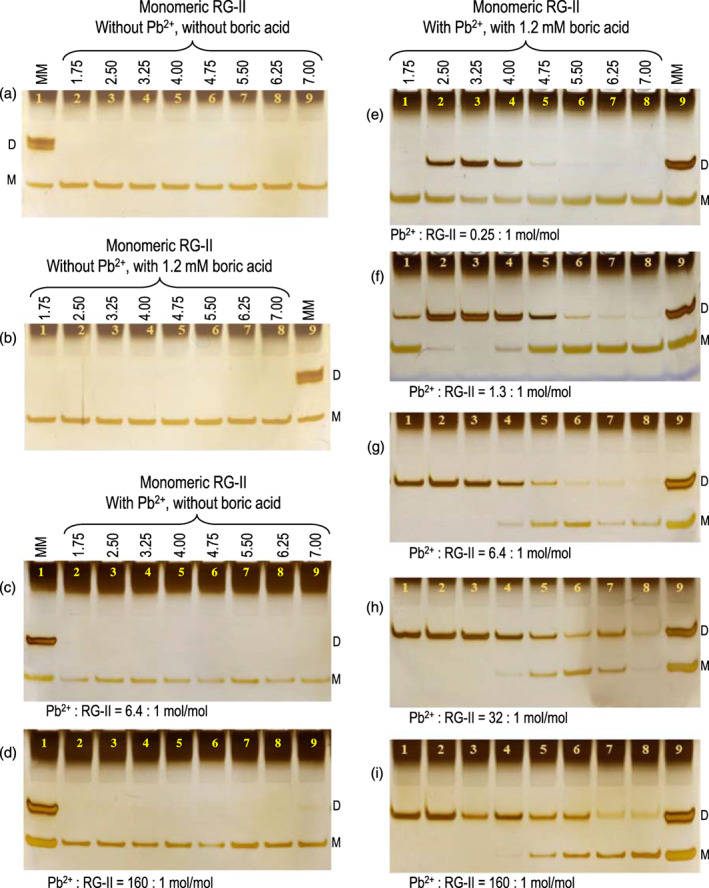
Effect of acidic pH on Pb^2+^/boron‐mediated rhamnogalacturonan‐II (RG‐II) dimerization. Reaction mixtures routinely contained 16 μm RG‐II monomer (80 μg ml^−1^), 1.2 mm boric acid, a 50 mm buffer, and various concentrations of lead nitrate. pH values (1.75–7.00) are shown above each set of related gels. (a,b) Gels show controls with no Pb^2+^, either (a) without or (b) with 1.2 mm boric acid. (c,d) Gels show controls with Pb^2+^ but without boric acid. After incubation at 20°C for 16 h, the products (equivalent to 0.8 μg RG‐II) were analysed by polyacrylamide gel electrophoresis. Marker‐mixture (MM) contained 0.8 μg each of RG‐II monomer (M) and dimer (D) obtained from de‐esterified *Rosa* AIR. (c)–(i) Pb^2+^/RG‐II molar ratios are shown at the bottom of gels. Absolute lead nitrate concentrations were: (c) 100 μm; (d) 2500 μm; (e) 4 μm; (f) 20 μm; (g) 100 μm; (h) 500 μm; and (i) 2500 μm. Buffers were: 50 mm threonine (Cl^−^, pH 1.75 and 2.50), formate (Na^+^, pH 3.25), acetate (Na^+^, pH 4.00, 4.75, and 5.50), MES (Na^+^, pH 6.25) or MOPS (Na^+^, pH 7.00).

‘Naked’ anionic RG‐II molecules are expected to repel each other electrostatically, hindering dimerization. As the *p*K_a_ of RG‐II is about 3.23, it will have only a negligible charge at pH 1.75. Therefore, if overcoming the electrostatic repulsion were sufficient to enable dimerization, we would expect to observe dimerization at pH 1.75, even in the absence of cationic chaperones. However, chaperone‐independent dimerization was undetectable at pH 1.75–7.00 (Figure 3a,b). (It will be shown below that dimerization at pH 1.75 is indeed possible in the presence of Pb^2+^.) Lower pH values were not tested as these would have quickly broken any newly formed boron bridges (e.g. at pH 1.0; Kobayashi et al., 1996; Matoh et al., 1993; O'Neill et al., 1996). We conclude that at all pH values tested here, boron bridging required the presence of RG‐II, boric acid, and a chaperone.

In the following work, we tested the ability of various cations in a range of acidic media (pH 1.75–7.0) to fulfil the proposed chaperoning role and thereby catalyse RG‐II dimerization *in vitro*. It should be noted that silver‐staining is more sensitive for the detection of dimeric RG‐II than of an equal mass of monomeric RG‐II (see figure 3a of Chormova et al., 2014). Thus, the extent of dimerization in different reaction mixtures may be easier to judge by looking at the disappearance of the monomer band rather than the formation of the dimer.

### Pb^2+^ as a chaperone: concentration and acid pH dependence

As reported before (Chormova et al., 2014; Ishii et al., 1999; O'Neill et al., 1996), Pb^2+^ was an excellent non‐biological chaperone, causing extensive boron bridging of RG‐II (Figure 3e–i). Pb^2+^ did not cause detectable dimerization in the absence of boron (Figure 3c,d). It was highly effective even at the lowest concentration tested (4 μm Pb^2+^ with 16 μm RG‐II; Figure 3e). Unlike with the peptide chaperones (see below), no *supra*‐optimal Pb^2+^ concentration was found (tested at up to 2500 μm; Figure 3i). The failure to reach a supra‐optimal concentration was not simply due to lead's limited solubility: we confirmed that, under all permutations tested in Figure 3 [pH 1.7–7.0, with 4–2500 μm Pb(NO_3_)_2_], the Pb^2+^ remained fully soluble with the single exception of 2500 μm Pb(NO_3_)_2_ at pH 7.00, which gave a white precipitate of Pb(OH)_2_ (Table S1). The additional presence of 1200 μm boric acid made little difference except that a weak precipitate now also formed in the mixture containing 2500 μm Pb(NO_3_)_2_ and 1200 μm boric acid at pH 6.8 as well as 7.0 (Table S1); indeed, lead borate is known to have limited solubility at neutral pH. Pb^2+^ was able to take RG‐II dimerization *to completion*, even at pH values as low as 1.75 (Figure 3f–i), proving that this extreme acidity does not prevent boron bridging (compare Figure 3b) – in contrast to the cleavage of boron bridges that occurs at pH 1.0 (HCl; Chormova et al., 2014; Kobayashi et al., 1996; Matoh et al., 1993; O'Neill et al., 1996).

When Pb^2+^ was the chaperone, no trimers or tetramers of RG‐II were detectable, in contrast to the effect of PH as chaperone under optimal conditions (see below).

Pb^2+^ ≥100 μm caused complete dimerization within 16 h at pH 1.75–3.25 (Figure 3g–i). On reducing the Pb^2+^ concentration to 4 or 20 μm to slow the boron bridging process, we found that the optimal pH for dimerization was 3.25 (Figure 3e,f).

Raising the pH from 3.25 to 7.00 led to progressively less dimerization at all lead concentrations. This would not be due to any ionic change in the boric acid, whose *p*K_a_ is 9.2, which is thus largely non‐ionic at all pH values tested (in the absence of sugars and alditols such as mannitol; Azevedo & Cavaleiro, 2012).

Regardless of the lead concentration and pH used, the electrophoretic mobilities of RG‐II monomer and dimer exactly matched those of the corresponding Pb‐free markers (MM lanes in Figure 3). This shows that the binding of lead to RG‐II was readily reversible, at least at the pH (8.8–9.1) of the electrophoresis system, with RG‐II–Pb and RG‐II–Pb–RG‐II complexes dissociating during electrophoresis (clearly shown in the absence of boron; Figure 3c,d). If Pb^2+^ had remained attached to RG‐II, the RG‐II's net charge would have become less negative, and its mass increased, diminishing the electrophoretic mobility. Thus, the role of lead in cross‐linking RG‐II can be described as catalytic.

**Figure 4:**
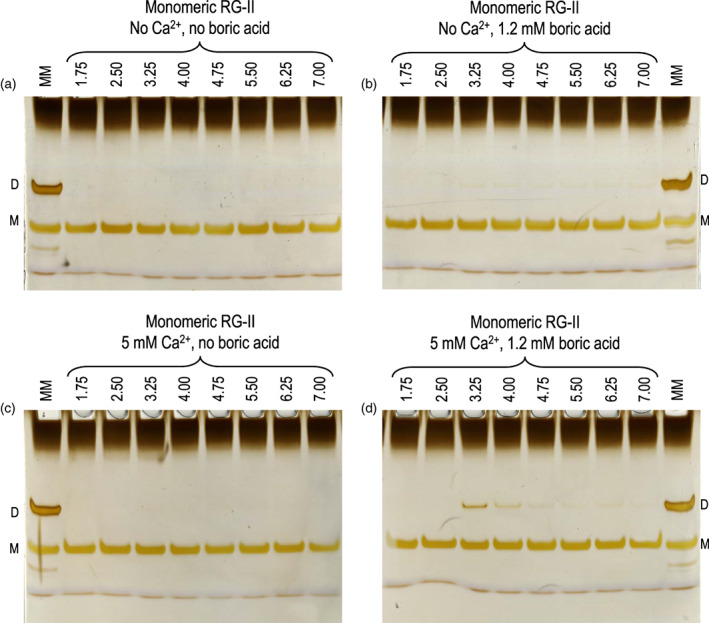
Effect of acidic pH on Ca^2+^/boron‐mediated rhamnogalacturonan‐II (RG‐II) dimerization. Reaction mixtures contained 16 μm RG‐II monomer (80 μg ml^−1^) and 50 mm buffer, plus (a) no further addition, (b) 1.2 mm boric acid, (c) 5 mm CaCl_2_, or (d) 1.2 mm boric acid plus 5 mm CaCl_2_. D, dimer; M, monomer. Other details as in Figure 3.

Ca^2+^ at 5 mm was much less effective as a chaperone than Pb^2+^ at 20 μm (Figure 4). As expected, no dimerization occurred with Ca^2+^ alone or with boric acid alone, regardless of pH (1.75–7.0), but 5 mm Ca^2+^ plus 1.2 mm boric acid brought about a low degree of dimerization with a pH optimum of 3.25.

In conclusion, Pb^2+^ even at a concentration as low as 4–20 μm strongly promoted the dimerization of RG‐II catalytically at pH 1.75 to about 4.75, and optimally at pH ≈ 3.25. Ca^2+^ also acted optimally at pH 3.25, but was far less effective than Pb^2+^.

### Peptide chaperones: concentration and acid pH dependence

**Figure 5:**
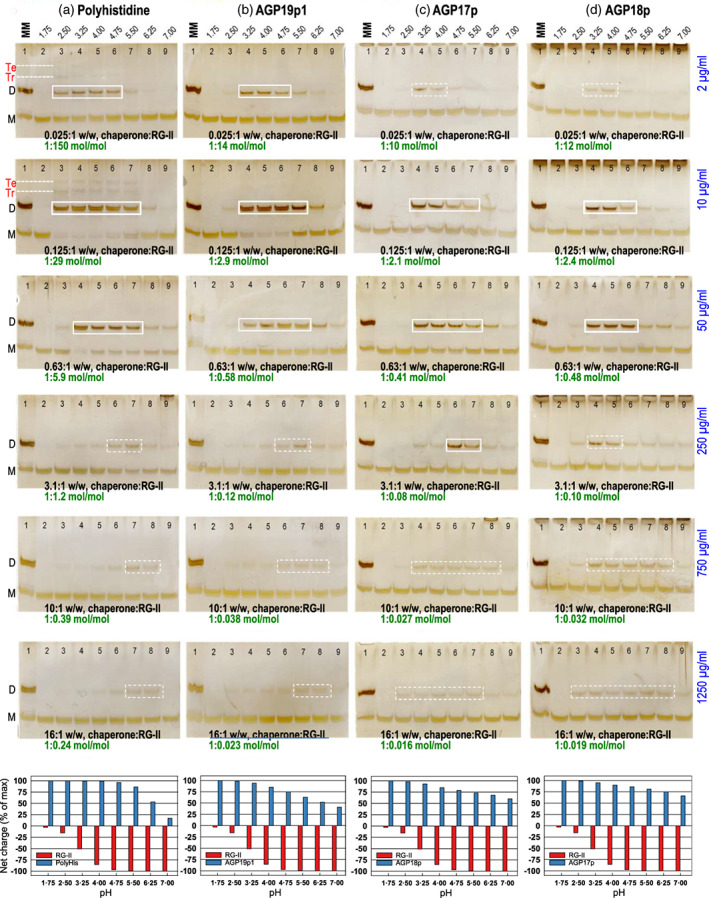
Effect of acidic pH on peptide‐mediated rhamnogalacturonan‐II (RG‐II) dimerization. Reaction mixtures contained 16 μm RG‐II monomer, 1.2 mm boric acid, a 50 mm buffer (listed in Figure 3), and the indicated chaperone: (a) polyhistidine; (b) AGP19p1; (c) AGP17p; and (d) AGP19p. pH values of the reaction mixtures are shown above each set of gels. Ratios shown at the bottom of each gel indicate the chaperone/RG‐II ratios (w/w and mol/mol); absolute chaperone concentrations were as shown on the right in blue. After incubation at 20°C for 16 h, the products (equivalent to 0.8 μg RG‐II) were analysed by polyacrylamide gel electrophoresis. Main dimer zones are highlighted by white boxes. Putative RG‐II trimer (Tr) and tetramer (Te) are labelled. Histogram below each set of gels shows the calculated effect of pH on the net charge of the chaperones (positive charges, blue bars) and RG‐II (negative charges, red bars). Marker‐mixture (MM, lane 1) contained 0.8 μg each of RG‐II monomer (M) and dimer (D), obtained from de‐esterified *Rosa* AIR. Net charges of peptides were estimated with IPC 2 (www.ipc2‐isoelectric‐point.org), which takes into account the influence of neighbouring amino acid residues on each other's ionization. It was assumed that an RG‐II monomer has 15 carboxy groups, with average pK_a_ ≈3.23. Charge on the imidazole side‐chain of His residue varies strongly with pH values above about 5. The α‐amino group of an oligopeptide and the side‐chains of Lys and Arg residues have pK_a_ values above 7, so they carry an almost full positive charge at all tested pH values. Each peptide also carries up to 1 negative charge (*p*K_a_ ≈3–4) at the C‐terminus.

All four cationic oligo‐/polypeptides tested (AGP17p, AGP18p, AGP19p1, and PH) promoted RG‐II dimerization in the presence of boric acid (Figure 5; the corresponding boron‐free controls are shown in Figure S3). Each exhibited a definite concentration optimum, with the lowest and highest concentrations tested causing less dimerization (Figure 5). Optimal peptide concentrations were 10–50 μg ml^−1^, irrespective of the peptide's molecular mass (1.3–18.4 kDa). These optimal chaperone concentrations equated to chaperone/RG‐II ratios of approximately 0.1:1 to 0.6:1 (w/w). The lowest highly effective concentration (10 μg ml^−1^) was approximately 0.5 and 7.8 μm for PH and AGP17p respectively, i.e. lower molar concentrations than of the substrates (16 μm RG‐II and 1200 μm boric acid). The effectiveness of a chaperone was thus catalytic, and largely determined by its w/v concentration rather than its molarity.

**Figure 6:**
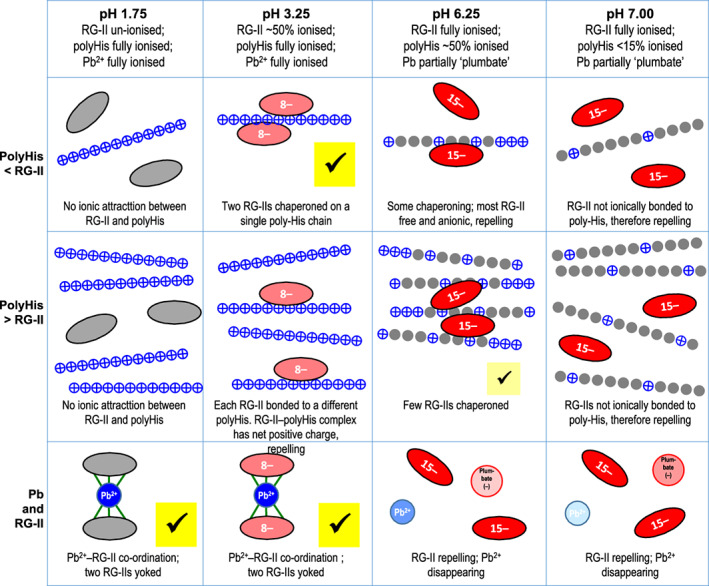
Interpretation of the effects of pH and cation concentration on rhamnogalacturonan‐II (RG‐II) dimerization. Oval, RG‐II molecule; chain of small circles, polyhistidine molecule; large circle, lead ion; green lines, co‐ordination bonds; red, negative charge; blue, positive charge; grey, little or no charge. A tick (✓) indicates that boron bridging occurs (small tick = slight boron bridging). A single Pb^2+^ ion is proposed to strongly cross‐link two RG‐II molecules by co‐ordination bonding. Cationic (poly)peptides are proposed to weakly chaperone RG‐II molecule(s) by ionic bonding.

The following description of the observations (Figure 5) is interpreted diagrammatically in Figure 6.

None of the peptides evoked any detectable dimerization in the most acidic medium tested (pH 1.75; Figure 5), even though Pb^2+^ was highly effective at that pH (Figure 3). This functional difference between Pb^2+^ and the peptides may reflect the ability of Pb^2+^ to form a co‐ordination complex between essentially non‐ionized RG‐II molecules at pH 1.75, whereas the cationic peptides are unable to form ionic bonds with non‐ionized RG‐II.

In the case of PH, at low chaperone/RG‐II ratios (0.03:1 to 0.6:1, w/w), the optimum pH for boron bridging was approximately 2.5–4.75 (Figure 5). Under these conditions, RG‐II is appreciably anionic and thus capable of forming ionic bonds with PH, which is almost fully (positively) ionized; see histograms in Figure 5. In this pH range, at the optimal chaperone/RG‐II ratio (approximately 0.025:1 to 0.125:1, w/w, i.e. with 2–10 μg ml^−1^ PH), monomeric RG‐II was moderately or highly dimerized within 16 h. In addition, at these PH concentrations, small amounts of two slower‐migrating RG‐II bands were produced, interpreted as trimer and tetramer. This result is in contrast to the effects of Pb^2+^, which produced only dimers (Figure 3).

On the other hand, with an excess of PH (chaperone/RG‐II ratios 3:1 to 16:1, w/w), RG‐II dimerization was severely diminished and the slight dimerization that did still occur peaked at a higher pH (approximately 5–6) where PH is incompletely ionized. At high PH concentrations, RG‐II dimerization was negligible both at low pH (<5) and at the highest pH tested (7.0) (Figure 5).

Compared with PH, the oligopeptide AGP19p1 more closely resembles a naturally occurring plant polycation. It was also an excellent chaperone, catalysing the boron bridging of RG‐II. Again, low chaperone/RG‐II ratios (between 0.025:1 and 0.6:1, w/w) promoted boron bridging optimally at pH 3.25–4.75 (Figure 5). At pH 4.0, AGP19p1 was almost as effective as PH, the optimal concentration being approximately 10 μg ml^−1^ for both these chaperones.

Unlike PH, the optimal concentration of AGP19p1 did not generate any detectable slower‐migrating RG‐II bands (putative trimer and tetramer), probably because the polycationic domain of a single AGP19p1 molecule is not large enough to seat more than two RG‐II molecules.

Like PH, AGP19p1 exhibited a supra‐optimal concentration; RG‐II dimerization occurred to a much lower extent with 250–1250 μg ml^−1^ AGP19p1 than with 10 μg ml^−1^ (Figure 5). Also resembling PH, the slight boron bridging that did still occur at high peptide concentrations was confined to the less acidic media (pH approximately 5–6).

AGP17p and AGP18p were also good chaperones, sharing many properties with AGP19p1 (Figure 5) in terms of concentration and pH optima and the inability to generate trimers and tetramers of RG‐II. However, one difference was that high concentrations of AGP17p and AGP18p did maintain some appreciable dimerization at the highest pH (7.0), whereas PH maintained none and AGP19p1 very little.

### Effect of ‘boron‐chelating’ sugars on the making and breaking of boron bridges

Sugars that occur principally in the furanose ring form and possess a *cis*‐diol group, such as apiose and ribose, are avid binders of borate (Hunt, 2002; Weigel, 1963). We argued that such sugars, by ‘chelating’ borate, could potentially split existing RG‐II boron bridges and/or prevent the formation of new ones. Even though never abundant *in vivo*, free ribose or apiose could be used pharmacologically to probe the boron bridging of RG‐II *in vivo*.

Adequate quantities of d‐apiose were obtained by mild acid hydrolysis of *Spirodela polyrhiza* (great duckweed) cell walls. A portion of the apiose obtained was assayed by thin‐layer chromatography (Figure S4). The yield of apiose obtained by this method was estimated by thymol staining to be 10 ± 2 μg per mg dry weight of alcohol‐insoluble residue (AIR). According to Pagliuso et al. (2018), the apiose content of *S. polyrhiza* was 5.42 ± 0.34 μg mg^−1^ dry weight. The stock contained negligible traces of contaminating fucose, arabinose, rhamnose and 2‐*O*‐methylxylose (Figure S4).

A potential strategy for investigating whether RG‐II dimerization occurs intraprotoplasmically or apoplastically could be to explore the effect of adding a membrane‐impermeant inhibitor of boron bridging. Here we show that the monosaccharide apiose is a candidate to serve this role.

An initial study tested ribose and apiose for their ability to remove boron from dimeric RG‐II and/or prevent the dimerization of monomeric RG‐II *in vitro*. Both these monosaccharides contain vicinal *cis*‐diol groups attached to a predominantly furanose ring structure (Figure S5), which is preferred for the formation of tetrahedral boron bridges (Bell et al., 1986; Chormova et al., 2014; Hunt, 2002; Kobayashi et al., 1996; O'Neill et al., 1996). Fucose was also tested, as it has been reported that diatoms and certain other algae having fucose‐rich cell walls require boron (Blevins & Lukaszewski, 1998) and because of the importance of fucosylation for the boron bridging of RG‐II, as demonstrated by fucose‐deficient *mur1‐1* and *sfr8* mutants (O'Neill et al., 2001; Panter et al., 2019).

Various concentrations of ribose, fucose, apiose, and apiin (an apiosylated flavonoid) were incubated in reaction mixtures containing either monomeric RG‐II (20 μm plus 1.2 mm boric acid and 2.7 μm PH) or dimeric RG‐II (10 μm). After 24 h at 20°C the products were analysed for dimerization. Under conditions that normally favour the boron bridging of RG‐II, apiose (100 mm) completely blocked dimerization (Figure S6, lanes 5 versus 13), and 1 mm apiin (lane 6) was moderately effective. Ribose and fucose had little effect, but 0.1 m HCl blocked dimerization (lane 10). As expected, dimerization was dependent on the presence of both boric acid and the chaperone (PH). We conclude that apiose and HCl can compete with boron–RG‐II bonding. However, none of the sugars tested was able to strip out the boron and break pre‐existing RG‐II dimers (Figure S6, lanes 22–27 versus 28).

**Figure 7:**
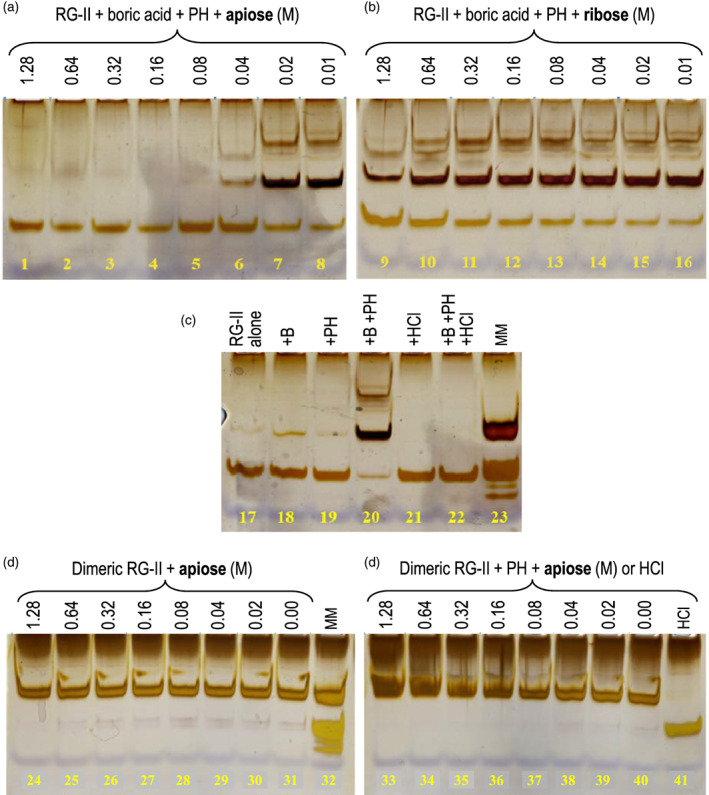
Concentrations of apiose and ribose blocking rhamnogalacturonan‐II (RG‐II) dimerization. All reaction mixtures were incubated at 20°C for 24 h and then analysed by polyacrylamide gel electrophoresis. Lanes 1–16 and 20: monomeric RG‐II (20 μm), boric acid (1.2 mm), polyhistidine (PH; 50 μg ml^−1^) and 50 mm acetate (Na^+^; pH 4.8) plus the sugars indicated (0.01–1.28 m apiose or ribose, or none). Lanes 17–19 and 21–22: similar mixtures (sugar‐free) modified to prevent dimerization. Lanes 24–31: reaction mixtures containing pre‐dimerized RG‐II plus the apiose concentrations indicated. Lanes 33–40: ditto but with 50 μg ml^−1^ PH. Lane 41: dimeric RG‐II monomerized with 0.1 m HCl. Lanes 23 and 32: marker mixture containing approximately 1 μg each of RG‐II monomer and dimer.

Apiose and ribose were next tested at a wider range of concentrations for prevention or reversal of dimerization (Figure 7). The minimum effective apiose concentration completely preventing RG‐II dimerization was 80 mm; 40 mm was also strongly effective. On the other hand, ribose only slightly inhibited dimerization at 1280 mm, the highest concentration tested. Again, apiose completely failed to monomerize previously boron‐bridged RG‐II dimers (Figure 7, lanes 24 and 33). This was equally true in the presence and absence of a cationic chaperone (PH).

**Figure 8:**
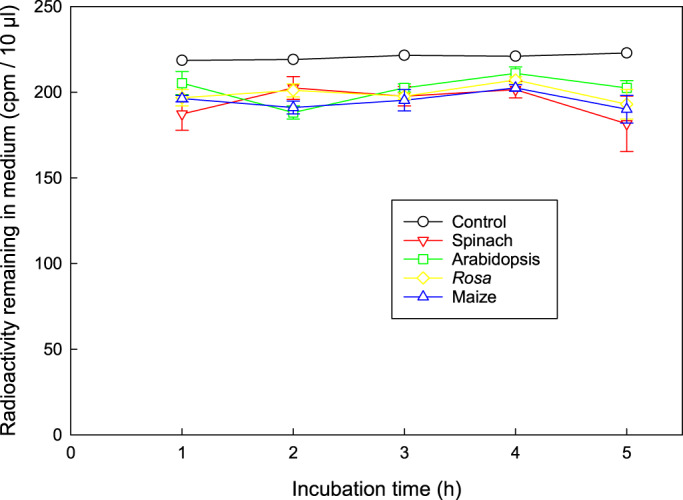
Time‐course of [^14^C]apiose feeding to cell‐suspension cultures. [U‐^14^C]Apiose (0.15 kBq; final concentration approximately 7 μm) was fed to 0.4 ml of 3‐day‐old axenic cell‐suspension cultures of spinach (carbon source glucose), Arabidopsis (carbon source glycerol), *Rosa* (glycerol), and maize (glucose) in flat‐bottomed vials with gentle shaking at 20°C. The control had no cells. Radioactivity remaining in the medium at intervals was assayed by scintillation counting.

Suspension‐cultured plant cells did not take up [^14^C]apiose from the medium during a 5‐h incubation when fed at approximately 7 μm (Figure 8). Therefore, an apiose supplement, employed to inhibit RG‐II dimerization *in vivo* (Figure 7), will be confined to the apoplast for at least 5 h, and thus not interfere with intraprotoplasmic RG‐II boron bridging. A 5‐h incubation is more than adequate to detect RG‐II dimerization, which can be observed within 4 min (Begum & Fry, 2022).

## DISCUSSION

### Dimeric RG‐II is stable under acidic conditions *in vivo* and *in vitro*

It has been known for many years that most of the RG‐II domains of pectin are bridged by borate diester linkages (Kobayashi et al., 1995; Kobayashi et al., 1996; Ishii et al., 1999; Matoh et al., 1993; O'Neill et al., 1996). These bridges are formed very rapidly after the synthesis of an RG‐II domain – mainly within the Golgi system, but also partly after its secretion into the apoplast (Begum & Fry, 2022; Chormova et al., 2014). However, we are not aware of any investigations into the longevity of boron bridges *in vivo*. Theoretically, boron‐starved plants could release their last remaining traces of boron from the dimeric RG‐II of old tissues and utilize the freed boric acid for the assembly of new cell walls in young tissues. A second reason why RG‐II might become monomerized *in vivo* would be to loosen the cell wall architecture, e.g. during acid growth. However, we found no evidence that boron‐starved or acid‐treated cells were able to cleave the boron bridges of dimeric RG‐II *in vivo* in media at pH 3.5–4.5. The experiment was done at the low pH values stated *and* in the presence of living cells; this environment would provide all relevant agents to achieve RG‐II monomerization even if low pH alone was inadequate to cleave boron bridges. This stability of the dimers thus shows that RG‐II dimerization is essentially irreversible *in vivo*, in cell‐suspension cultures tested.

We tested more extreme acidity *in vitro*. RG‐II dimers are monomerized *in vitro* at pH 1.0 (in 1 h at 20°C), without hydrolysing glycosidic bonds (Chormova et al., 2014; Ishii et al., 1999; O'Neill et al., 1996). However, we found that boron bridges were not cleaved *in vitro* at any of a wide range of ‘apoplastic’ pH values, down to pH 2, at 20°C. This finding might question the significance of reports that Ca^2+^ stabilizes the boron‐bridged dimer of RG‐II (Kobayashi et al., 1999; Matoh & Kobayashi, 1998) as we found that the dimer is very stable at all tested pH values (≥2) even in the absence of divalent cations.

RG‐II contains some very acid‐labile glycosidic bonds, particularly the β‐d‐apiosyl‐(1 → 2)‐d‐galacturonate linkages. The latter are reported to be 35% hydrolysed within 24 h in 0.1 m trifluoroacetic acid (pH ≈ 1.1) at 40°C (Spellman et al., 1983), from which one could predict roughly 10% apiosyl bond hydrolysis in 240 h at pH 2 and 20°C (the conditions used in Figure 2), and thus hydrolysis of approximately 20% of the monomeric RG‐II molecules, as there are two apiose residues per RG‐II monomer. However, RG‐II was not appreciably lost by glycosidic bond hydrolysis under the most acidic conditions we tested.

In conclusion, RG‐II is chemically stable – both its glycosidic linkages and its boron bridges – even under the most severely acidic conditions likely to be encountered in the apoplast of a growing plant cell. It is remarkable that cell expansion evidently occurs without breaking any of the numerous existing, permanent, covalent RG‐II–RG‐II interconnections in the primary cell‐wall architecture.

### RG‐II dimerization *in vitro* requires a suitable cationic ‘catalyst’, even in the absence of electrostatic repulsion

Simply mixing RG‐II with boric acid in aqueous solution does not result in appreciable boron bridging (Figures 3b and 4b). On the contrary, a positively charged chaperone such as Pb^2+^ or a cationic peptide must also be present (Chormova & Fry, 2016; Ishii et al., 1999; O'Neill et al., 1996; Sanhueza et al., 2022). Such chaperones were assumed to work by cancelling the negative charges of RG‐II such that two anionic polysaccharide molecules could approach each other closely enough for boron bridging. This interpretation, however, must be an oversimplification as we found that a chaperone is still required for RG‐II dimerization even at pH 1.75, where an RG‐II monomer carries only a slight charge (about −0.5 of a maximum of about −15) and thus electrostatic repulsion would be minimal. We conclude that neutralizing the negative charge on RG‐II is not sufficient to enable boron bridging; most likely a mechanism needs to be in place to bring two RG‐II molecules actively into close proximity. The following considerations suggest that Pb^2+^ and cationic peptides achieve this via different mechanisms.

### Pb^2+^ as a chaperone: effect of pH

Lead nitrate at 4–2500 μm catalysed excellent boron bridging of RG‐II at pH 1.75–3.25. Importantly, this shows that RG‐II dimerization is possible at pH values as low as 1.75, which is close to the pH (1.0) routinely used in the laboratory to monomerize dimeric RG‐II.

At the lowest Pb^2+^ concentration tested (4 μm, i.e. 1 Pb^2+^ ion per 4 RG‐II molecules), the optimum pH for dimerization was 3.25. At pH values of ≥4.75, 20–500 μm lead nitrate became only weakly effective as a chaperone, even though these conditions did not cause any of the lead to precipitate as lead hydroxide or lead borate (Table S1). Such precipitation occurred only when the initial lead nitrate concentrations was greater than 500 μm, and then only at pH ≥6.8 (Table S1). Thus, the loss of effectiveness of lead as a chaperone as the pH was raised to ≥4.75 is not principally due to precipitation of lead. Alternatively, the loss of effectiveness of lead nitrate with rising pH could be due to:

- RG‐II becoming almost fully negatively charged (see histograms in Figure 5) and thus too mutually repulsive for Pb^2+^ to be able to bring two RG‐II molecules together for subsequent boron bridging;
- conversion of some of the Pb^2+^ to ‘plumbates’, e.g. Pb(OH)_6_^2−^, which are not cationic. However, even at pH values as high as 6.5, the equilibrium still favours of lead‐based cations, as shown by comparing the electrophoretic mobility of Pb [initially as Pb(NO_3_)_2_] at pH 2.0 (all Pb^2+^) versus 6.5 (less mobile but still net cationic; figure 5b,c of Fry, 2017). This is also supported by Wang et al. (2009), who report Pb^2+^ and PbOH^+^ predominating at pH 6.5–7.5, although they do not mention any anionic ‘plumbates’ except Pb(OH)_3_^−^ (only forming at pH values >10). Therefore, adequate Pb^2+^ ion concentrations will have been present at all pH values tested.

Explanation (i) above is therefore preferred.

### Pb^2+^ as a chaperone: effect of concentration

The lowest Pb^2+^ concentration tested (4 μm) corresponded to a molar Pb/RG‐II ratio of 0.25:1, thus far too few Pb^2+^ ions to cancel the net charge (−15) of RG‐II at most pH values tested. It appears more likely that the Pb^2+^ complexes with RG‐II via co‐ordinate bonds to bring together two RG‐II molecules, as RG‐II–Pb^2+^–RG‐II, closely enough for boron bridging. Indeed, Pb^2+^ is known to form co‐ordinate bonds with many carbohydrates, particularly pyranose residues with three –OH groups in axial–equatorial–axial configuration or furanose residues with two –OH groups in a *cis–cis* sequence, e.g. apiose (Angyal, 1973; Angyal & Davies, 1971; Ul'yanovskii et al., 2022).

O'Neill et al. (1996) showed that Pb^2+^, Sr^2+^, and Ba^2+^ form long‐lived co‐ordination complexes with dimeric RG‐II, which are not easily disrupted by chelation (specifically, treatment with Chelex® resin). However, we found no evidence for any effect of Pb^2+^ on the electrophoretic mobility of either dimeric or monomeric RG‐II, suggesting that RG‐II–Pb^2+^–RG‐II and Pb^2+^–RG‐II complexes are unstable under the conditions of polyacrylamide gel electrophoresis (PAGE), which is alkaline (gel pH 8.8; electrode buffer pH 9.1).

At all pH values tested, raising the lead concentration from 20 μm (1.25 Pb^2+^ ions per monomeric RG‐II molecule; Figure S3c) to 2500 μm (156 Pb^2+^ ions per RG‐II) never appreciably decreased RG‐II dimerization, in contrast with the peptide chaperones. Therefore, it cannot be argued that high lead created Pb^2+^_*n*_–RG‐II complexes that carried a net positive charge and thus again mutually repelled, preventing dimerization.

### Peptide chaperones

Pb^2+^ cannot be the natural chaperone responsible for RG‐II dimerization *in vivo* because Pb is not an essential element for plants. Therefore, to explore more natural processes, we characterized the chaperone function of cationic peptides. We tested one wholly artificial polypeptide, PH (mean degree of polymerization ≈ 100), as well as three more natural candidates: highly cationic oligopeptide fragments occurring within the sequences of Arabidopsis AGP17, 18, and 19. Similar peptide sequences are also known from numerous diverse land plants (Baldwin et al., 2001; Gong et al., 2012), including lycopodiophytes (onekp|PQTO_scaffold_2013256_Lycopodium_deuterodensum.2).

The highly cationic domains might theoretically be shielded from RG‐II in the mature AGPs, which could be indicated by 3D structures. To date, only low‐confidence predictions are available for the 3D structures of AGP17, 18, and 19 (https://alphafold.ebi.ac.uk/entry/O22194, /Q9FPR2, and /Q9S740 respectively). Nevertheless, these predictions show the highly cationic portions of each of these AGPs to be exposed, such that they may be accessible to RG‐II. The predictions do not include the glyco‐moieties of the AGPs; however, there are no glycosylation sites within the highly cationic stretches (figure 6 of Sun et al., 2005), and therefore we suggest that glycosylation does not shield the AGPs from ionic interaction with RG‐II.

### PH can generate RG‐II trimers and tetramers

PH was the most effective peptide chaperone tested, catalysing essentially complete cross‐linking of RG‐II under optimal conditions (e.g. 10 μg ml^−1^ at pH 3.25; Figure 5a). Interestingly, the products formed under these conditions included not only the dimer but also probable trimers and tetramers. Forming an RG‐II trimer through boron bridging of apiose residues must mean that at least one of the two boron bridges involved side‐chain B, which bears the less favourable of the two apiose residues in an RG‐II molecule. If we assume (as reported by Ishii et al., 1999; O'Neill et al., 1996; Pellerin et al., 1996; Shimokawa et al., 1999) that the single boron bridge of an RG‐II dimer is between side‐chain A of each partner, then the dimer can be represented as

$${\mathrm{Api}}^{B}‐❶‐{\mathrm{Api}}^{A}>\left[B^{-}\right]<{\mathrm{Api}}^{A}‐❷‐{\mathrm{Api}}^{B}$$

where ➊ and ➋ are the two participating RG‐II monomers, Api^A^ and Api^B^ are the apiose residues of side‐chains A and B, and >**[B** ^**‐**^ **]**< is the anionic tetrahedral boron atom of the borate diester bridge.

Thus, the trimer could be

$${\mathrm{Api}}^{B}‐❶‐{\mathrm{Api}}^{A}>\left[B^{-}\right]<{\mathrm{Api}}^{A}‐❷‐{\mathrm{Api}}^{B}>\left[B^{-}\right]<{\mathrm{Api}}^{A}‐❸‐{\mathrm{Api}}^{B}$$

or possibly

$${\mathrm{Api}}^{B}‐❶‐{\mathrm{Api}}^{A}>\left[B^{-}\right]<{\mathrm{Api}}^{A}‐❷‐{\mathrm{Api}}^{B}>\left[B^{-}\right]<{\mathrm{Api}}^{B}‐❸‐{\mathrm{Api}}^{A}$$

and the tetramer could be

$${\mathrm{Api}}^{B}‐❶‐{\mathrm{Api}}^{A}>\left[B^{-}\right]<{\mathrm{Api}}^{A}‐❷‐{\mathrm{Api}}^{B}>\left[B^{-}\right]<{\mathrm{Api}}^{A}‐❸‐{\mathrm{Api}}^{B}>\left[B^{-}\right]<{\mathrm{Api}}^{A}‐❹‐{\mathrm{Api}}^{B}$$

or similar.

It is possible that boron bridges involving side‐chain B are less stable and are thus difficult to isolate from plant cell walls. Indeed, we were unable to isolate the trimers and tetramers intact by elution from a polyacrylamide gel; when re‐electrophoresed, the eluted ‘trimers’ and ‘tetramers’ showed only RG‐II dimers.

A PH molecule has a length of approximately 16 nm and a net charge (at pH 3.25) of about +100. Thus, at low concentration, a single PH molecule could simultaneously anchor more than two RG‐II molecules (each approximately 4 nm), which we propose to be the basis of the formation of trimers and tetramers of RG‐II. Unlike PH, optimal concentrations of the AGP oligopeptides did not generate any detectable trimers or tetramers (Figure 5b–d), presumably because the polycationic domain of the AGP oligopeptides (approximate length 2 nm) is not large enough to seat more than two RG‐II molecules simultaneously.

### Peptide chaperones: pH dependence

The optimum pH for RG‐II dimerization was 3.25–4.00 for all peptide chaperones tested (including PH). This is attributed to the peptides' almost full ionization and the RG‐II's partial ionization within this pH range (histograms, Figure 5), causing a strong ionic interaction between RG‐II and the peptide. Of the three AGP oligopeptides, high concentrations of AGP17p and AGP18p maintained appreciable dimerization at the highest pH tested (pH 7), whereas PH maintained none at that pH and AGP19p1 very little. This may relate to the fact that AGP17p and AGP18p owe their cationic properties almost entirely to Lys residues, which are almost fully ionized up to (and above) pH 7. On the other hand, AGP19p1 possesses six His residues and therefore decreases drastically in charge as the pH rises to 7, as does PH. We estimate that at pH 7, AGP17p and AGP18p are still approximately 60–65% fully cationic (histograms in Figure 5) whereas AGP19p1 and PH are only approximately 40% and 17% fully cationic respectively.

### Peptide chaperones: concentration dependence

High concentrations of the peptide chaperones strongly diminished RG‐II dimerization. This is interpreted as because, at a high peptide concentration:

- only rarely do two RG‐II molecules become ionically bonded to a single peptide molecule and thus drawn close enough together for boron bridging to occur;
- the RG‐II–peptide complexes (with an excess of peptide) will have a net positive charge, thus mutually repelling each other electrostatically.

When the RG‐II concentration exceeds the chaperone on a w/w basis (i.e. in these experiments, when the chaperone concentration is ≤50 μg ml^−1^), two or more RG‐II molecules are likely to become crowded on the same peptide molecule, and thus brought into sufficiently close contact for boron bridging. With PH as the chaperone, more than two RG‐II molecules can be bonded to a single PH molecule, making it possible to produce trimers and tetramers of RG‐II, as observed, in addition to dimers. The oligopeptides, on the other hand, are only big enough to accommodate two RG‐II molecules – resulting in dimers but not trimers or tetramers. The crowding of RG‐II molecules on a single AGP cationic oligopeptide might be even more constrained in the native AGP glycoprotein, where the neighbouring arabinogalactan side‐chains would limit accessibility.

### Cationic peptides and divalent metal ions achieve RG‐II dimerization via different mechanisms: ionic versus co‐ordinate bonding

The results show that simply neutralizing the negative charge on RG‐II does not of itself facilitate boron bridging in the absence of cationic chaperones. Thus, it is clear that a mechanism needs to be in place to bring together two RG‐II molecules proactively, rather than merely ensuring the absence of their mutual electrostatic repulsion. Our work shows that Pb^2+^ and cationic peptides achieve this by different mechanisms.

Pb^2+^ works with just 1.25 metal ions (each with a charge of +2) per RG‐II molecule (charge about −8 at pH 3.25). This quantity of Pb^2+^ clearly cannot overcome the net negative charge of RG‐II to prevent their electrostatic repulsion. Instead, a single Pb^2+^ ion (diameter approximately 0.8 nm in aqueous solution) must firmly cross‐link two much larger (approximately 4 nm based on the length of the homogalacturonan backbone; Walkinshaw & Arnott, 1981), and more highly charged RG‐II molecules, bringing them within reach for boron bridging. The few, but strong, RG‐II–Pb^2+^–RG‐II linkages are proposed to be co‐ordinate bonds rather than ionic bonds. For example, Pb^2+^ can bond to β‐d‐glucuronic acid (Tajmir‐Riahi, 1989), a constituent residue of RG‐II. We assume that Ca^2+^ (Figure 4) works in a similar manner to Pb^2+^, although less effectively.

On the other hand, the cationic oligopeptides have numerous positively charged groups. At pH 3.25, the net charges of AGP17p, AGP18p, and AGP19p1 are +10, +10, and +12 respectively, compared with about −8 for RG‐II. The length of an AGP19p1 chain is roughly 2 nm, which is apparently large enough to anchor simultaneously two approximately 4‐nm RG‐II molecules, possibly one on each side of the peptide.

### Possible synergy of co‐ordinate and ionic bonding for RG‐II dimerization *in vivo*

Pb^2+^ cannot be a natural agent of RG‐II dimerization *in vivo*. Ca^2+^ also catalyses RG‐II dimerization *in vitro* (Figure 4d) but only weakly except at unphysiologically high concentrations. We suggest that less effective, but biologically relevant, divalent metal ions such as Ca^2+^ at low concentration may contribute to the *in‐vivo* cross‐linking of two RG‐II molecules via co‐ordinate bonding rather than principally via ionic bonding. Ca^2+^–sugar coordinate bonds are described by, for example, Tajmir‐Riahi (1986) and Yang et al. (2002). Their existence is also indicated by the fact that Rezex/Ca^2+^ high‐performance liquid chromatography columns give strongly different sugar separations from Rezex/H^+^ columns, indicating sugar interactions with Ca^2+^; in particular, ribose binds very well to Rezex/Ca^2+^, and apiose (another furanose sugar with a *cis*‐diol) may behave similarly.

The Ca^2+^ would become more effective *in vivo* when synergized by ionic bonding of the RG‐II to polycationic peptide chaperones naturally occurring in the apoplast. It will be of interest in future experiments to test Ca^2+^/peptide synergy in RG‐II dimerization.

It is possible that some of the arabinogalactan side‐chains of native AGP17, 18, and 19, possess a Ca^2+^‐(glucuronate)_2_ unit (Lamport & Várnai, 2013) optimally positioned to transfer Ca^2+^ to form a co‐ordinate bond with the RG‐II molecules that are ionically bonded to the adjacent highly cationic domain (Figure S7).

### Effect of apoplastic ‘boron‐chelating’ sugars on the making and breaking of boron bridges

*In‐vivo* experiments to explore the subcellular location of RG‐II dimerization would benefit from an agent that blocks boron bridging specifically in the apoplast and not in the Golgi system. In this work, we report that free apiose at ≥40 mm blocks RG‐II dimerization and that exogenous apiose is not taken up by plant cells, hence fulfilling the above criteria. Free apiose, even at concentrations >1 m, does not cleave pre‐existing borate diester bonds in RG‐II. Thus, exogenous 40–80 mm apiose is a pharmacological tool specifically inhibiting apoplastic RG‐II dimerization.

We reasoned that apiose might on the other hand promote the cleavage of boron bridges if assisted by a chaperone such as PH. Theoretically, PH, which is known to enhance the formation of boron bridges, might also enhance the reverse, in an equilibrium:

$$\mathrm{PH}•{\left(\mathrm{RG}‐\mathrm{II}\right)}_{2}+H_{3}{\mathrm{BO}}_{3}↔\mathrm{PH}•\left(\mathrm{RG}‐\mathrm{II}>\left(B^{-}\right)<\mathrm{RG}‐\mathrm{II}\right),$$

thus, aiding any ability of monomeric apiose to strip boron out of the dimer. However, this hypothesis was not supported by the data, and no ability of apiose to ‘chelate’ boron out of dimeric RG‐II could be demonstrated even in the presence of peptide chaperones (Figure 7).

Therefore, an apiose supplement, employed to inhibit RG‐II dimerization *in vivo*, will be confined to the apoplast and can be used to test for intraprotoplasmic boron bridging, which would continue in the presence of apoplastic apiose. Prevention of dimerization by exogenous apiose would indicate that RG‐II dimerization can occur post‐secretion; but if apiose had no effect on dimerization, this would indicate that dimerization occurs in a subcellular compartment (e.g. within Golgi bodies or secretory vesicles) inaccessible to apoplastic apiose.

## CONCLUSION

In conclusion, an acidic (‘apoplastic’) pH does not affect pre‐existing boron bridges in dimeric RG‐II, either *in vivo* or *in vitro*. Thus, auxin‐induced ‘acid growth’ is not achieved by pH‐dependent modification of RG‐II cross‐linking. In addition, our data indicate that boron‐starved cells cannot re‐mobilize residual boron from its attachment to dimeric RG‐II. Divalent metal ions and cationic peptides catalyse boric acid‐dependent dimerization by transiently attaching to RG‐II via co‐ordinate and ionic bonds respectively. This study demonstrates that levels of acidity similar to those of Golgi cisternae and vesicles, and in the apoplast, are compatible with new RG‐II dimerization. The nature of the interaction between monomeric RG‐II and the cationic chaperones, and their molar ratio, are important for successful dimerization of RG‐II.

## EXPERIMENTAL PROCEDURES

### Maintenance of cell‐suspension cultures

Cell‐suspension cultures of *Rosa* sp. (‘Paul's Scarlet’ rose; a complex hybrid) were grown at 25°C under constant dim illumination (approximately 10 μmol m^−2^ sec^−1^) with orbital shaking at approximately 114 r.p.m. in a medium containing 3.3 μm boric acid (Fry & Street, 1980) with the usual carbon source replaced by 2% glycerol. [Glycerol media help to promote uptake of added traces of sugars (Sharples & Fry, 2007), but glycerol is not expected to make any difference to RG‐II behaviour.] The cultures were grown at 50 ml per 250‐ml glass flask and subcultured fortnightly. For some experiments, boric acid was omitted from the medium and these cultures were grown in polycarbonate flasks to avoid contamination with boron solubilized from glass flasks (Chormova et al., 2014).

The other species of cell cultures were as described previously: Arabidopsis (*Arabidopsis thaliana* L.; Sharples & Fry, 2007), spinach (*Spinacia oleracea* L., ‘Monstrous Viroflay’; Dalton & Street, 1976), and maize (*Zea mays* L., ‘Black Mexican sweetcorn’; Kerr & Fry, 2003).

### Preparation of RG‐II

Cultured *Rosa* cells (grown with or without 3.3 μm boric acid in the medium) were rinsed in water, then AIR was prepared by stirring in two changes of 75% (v/v) ethanol at 20°C, each for 4–6 h. Pectin in the AIR was de‐esterified with 1 m Na_2_CO_3_ at 4°C for 16 h, then the AIR suspension was slightly acidified with acetic acid, rinsed with water until neutral and freeze‐dried. Endopolygalacturonase (10 U ml^−1^; Megazyme, Bray, Co. Wicklow, Ireland;  http://www.megazyme.com/) was added (approximately 50 μl mg^−1^ AIR) and incubated at 20°C for 16 h in 50 mm acetate (Na^+^, pH 4.8). The solubilized RG‐II (dimeric and monomeric, from +B and −B cultures respectively) was purified by gel‐permeation chromatography on Bio‐Gel P‐30 (Bio‐Rad, Irvine, CA, USA) in pyridine/acetic acid/water (1:1:98, pH 4.7, containing 0.5% chlorobutanol), then dried *in vacuo*.

### Chaperones

Poly‐l‐histidine (Cl^−^ salt), an artificial polypeptide composed of histidine residues (mean degree of polymerization approximately 106; approximately 14.6 kDa [or 18.4 kDa, counting the Cl^−^ ions], pI ≈ 7.8), was from Sigma‐Aldrich (Poole, Dorset, UK).

Highly basic oligopeptide fragments of Arabidopsis AGP17, AGP18, and AGP19,

- AGP17p (KHKKKTKKHK, 1.29 kDa, pI ≈ 8.2),
- AGP18p (KHKKTTKKSKKH, 1.48 kDa, pI ≈ 8.2), and
- AGP19p1 (KHKRKHKHKRHHH, 1.79 kDa, pI ≈ 8.8),

were synthesized by Thermo Fisher Scientific (Inchinnan, Renfrew, UK). The approximate pI values were predicted by IPC 2.0 (www.ipc2‐isoelectric‐point.org). Inorganic chaperones (lead nitrate and calcium chloride) were from Sigma‐Aldrich.

### Preparation of apiose

d‐Apiose was prepared from *S. polyrhiza*, which is rich in apiogalacturonan (Grisebach, 1980), from which apiose is readily released by mild acid hydrolysis. *S. polyrhiza* plants were washed, homogenized and acid hydrolysed in 0.67 m trifluoroacetic acid at 100°C for 1 h. The acid hydrolysate was paper chromatographed in butan‐1‐ol/acetic acid/water 12:3:5 containing 0.55% w/v phenylboronic acid (PBA). The fast‐migrating zone containing the apiose–PBA complex was cut out and eluted with 1 m formic acid. The eluate was phase‐partitioned between 1 m formic acid (aqueous) and butan‐1‐ol; the aqueous phase containing the PBA‐free apiose was dried and re‐dissolved in water, and a portion was analysed by thin‐layer chromatography on silica‐gel plates in ethyl acetate/pyridine/acetic acid/water (6:3:1:1) (Franková & Fry, 2011) with thymol/H_2_SO_4_ staining (Jork et al., 1994).

### Preparation of [U‐^14^C]apiose

Growing *Lemna minor* fronds were placed in a 250‐ml screw‐top flask with 100 ml of autoclaved, filtered pond water. Carrier‐free NaH^14^CO_3_ (8 MBq) was added to the solution, and the flask was immediately capped and incubated under a xenon arc lamp for 7 days. The fronds were then washed in 75% ethanol containing 1% acetic acid followed by 75% ethanol four times, and the AIR (approximately 20 mg) was hydrolysed with 0.1 m trifluoroacetic acid at 80°C for 1 h. The hydrolysate was run by preparative paper chromatography (butan‐1‐ol/acetic acid/water, 12:3:5, 17 h) on Whatman No. 3 paper and the radioactive sugars were visualized by autoradiography. The radioactive band that co‐migrated with an apiose marker was eluted with water; yield 68 kBq (specific activity approximately 52 kBq/μmol).

### Feeding exogenous dimeric RG‐II to living cultures

Purified dimeric RG‐II was sterilized by drying from a small volume of 75% ethanol under a stream of sterile air, then dissolved in sterile water and added to a 300‐μl aliquot (‘mini‐culture’) of 3‐day‐old zero‐boron *Rosa* cell suspension (15% packed cell volume, fresh weight approximately 52 mg, medium unbuffered [pH ≈ 5.2]) to give 26 μm final dimer concentration. The mini‐cultures were incubated under standard conditions in round‐bottomed polypropylene tubes (internal diameter 12 mm) capped with cotton wool. Controls received no exogenous RG‐II. Replicate mini‐cultures were harvested at intervals and aliquots of spent medium were analysed by PAGE. After a 96‐h incubation the cells were tested for viability. In other experiments, the mini‐cultures were buffered with 25 mm succinate (Na^+^) to pH 3.5, 4.0, or 4.5.

### Fate of monomeric and dimeric RG‐II at various acidic pH values *in vitro*

To study the stability of RG‐II *in vitro*, we incubated pure RG‐II (26 μm monomer or 13 μm dimer) in 25 mm pyridine buffers (formate pH 2.0–3.0, or acetate pH 3.5–7.0) for 0.5–240 h. Samples were then dried, removing the volatile buffers, and analysed by PAGE.

To study the dimerization of RG‐II *in vitro*, we incubated 16 μm monomeric RG‐II in 50 mm buffer (pH 1.75–7.0; for details see figure legends) with and without 1.2 mm boric acid and various concentrations of assorted cationic chaperones (for w/v and molar concentrations, see Table S2).

### Polyacrylamide gel electrophoresis

RG‐II samples were analysed by PAGE, and fixed and stained as described by Sanhueza et al. (2022).

### Assessing cell viability

Cultured cells were bathed in 2% Evan's blue for 1–2 min, then rinsed in water and observed microscopically. This reagent stains the protoplasm of dead cells.

### Testing uptake of [ ^14^C]apiose by suspension‐cultured plant cells

[U‐^14^C]Apiose (0.15 kBq) was fed to 3‐day‐old axenic cell‐suspension cultures (0.4 ml, thus final apiose concentration ≈7 μm) of spinach, Arabidopsis, rose, and maize in flat‐bottomed vials with gentle shaking at 20°C. Uptake was monitored hourly by sampling the medium.

## AUTHOR CONTRIBUTIONS

SCF planned the study, SCF and RAB designed the study, RAB and DJM performed the experiments, SCF and RAB prepared the figures and drafted the manuscript; and SCF edited them. All authors approved the manuscript.

## CONFLICT OF INTEREST

The authors declare that they have no competing interests.

## Supporting information


**Figure S1.** Evan's blue staining of *Rosa* cells after 96 h incubation with exogenous dimeric RG‐II in zero‐boron medium, pH unadjusted.
**Figure S2.** Evan's blue staining of *Rosa* cells after 96 h in zero‐boron acidic medium, pH adjusted to 3.5–4.5.
**Figure S3.** Boron‐free controls for studying the effect of acidic pH on chaperone‐mediated RG‐II dimerization.
**Figure S4.** Quantification of apiose in stock solution by TLC.
**Figure S5.** Structure of d‐ribose and d‐apiose.
**Figure S6.** Testing sugars for ability to prevent or reverse RG‐II dimerization *in vitro*.
**Figure S7.** *At*AGP19 protein sequence and glycosylation.

**Table S1.** Effect of pH on solubility of Pb^2+^ with and without boric acid.
**Table S2.** Typical composition of *in‐vitro* reaction mixtures with monomeric RG‐II, boric acid, and cationic chaperones to study dimerization.

## Data Availability

All relevant data can be found within the manuscript and its supporting material.
