# Supplementary material for: Making and breaking of boron bridges in the pectic domain rhamnogalacturonan‐II at apoplastic pH in vivo and in vitro
Source: Plant J. 2023 Feb 8;113(6):1310–29. doi: 10.1111/tpj.16112 (PMC10952590; doi:10.1111/tpj.16112)
Supplement: Supplementary file 1 — Figure S1. Evan's blue staining of Rosa cells after 96 h incubation with exogenous dimeric RG‐II in zero‐boron medium, pH unadjusted. Figure S2. Evan's blue staining of Rosa cells after 96 h in zero‐boron acidic medium, pH adjusted to 3.5–4.5. Figure S3. Boron‐free controls for studying the effect of acidic pH on chaperone‐mediated RG‐II dimerization. Figure S4. Quantification of apiose in stock solution by TLC. Figure S5. Structure of d‐ribose and d‐apiose. Figure S6. Testing sugars for ability to prevent or reverse RG‐II dimerization in vitro. Figure S7. AtAGP19 protein sequence and glycosylation. [file TPJ-113-1310-s002.pptx]

## Slide 1
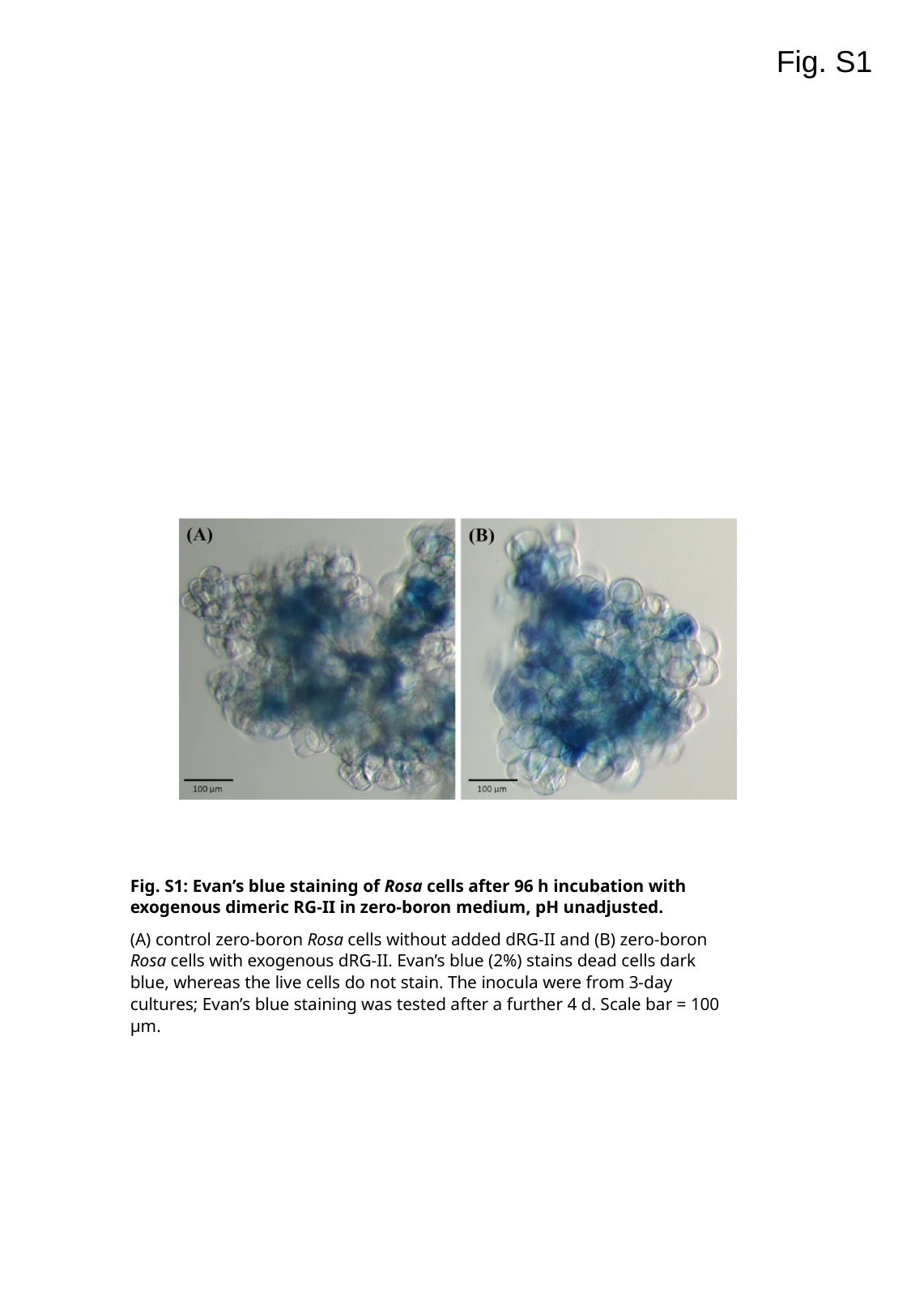

Fig. S1
Fig. S1: Evan’s blue staining of Rosa cells after 96 h incubation with exogenous dimeric RG-II in zero-boron medium, pH unadjusted.
(A) control zero-boron Rosa cells without added dRG-II and (B) zero-boron Rosa cells with exogenous dRG-II. Evan’s blue (2%) stains dead cells dark blue, whereas the live cells do not stain. The inocula were from 3-day cultures; Evan’s blue staining was tested after a further 4 d. Scale bar = 100 µm.

## Slide 2
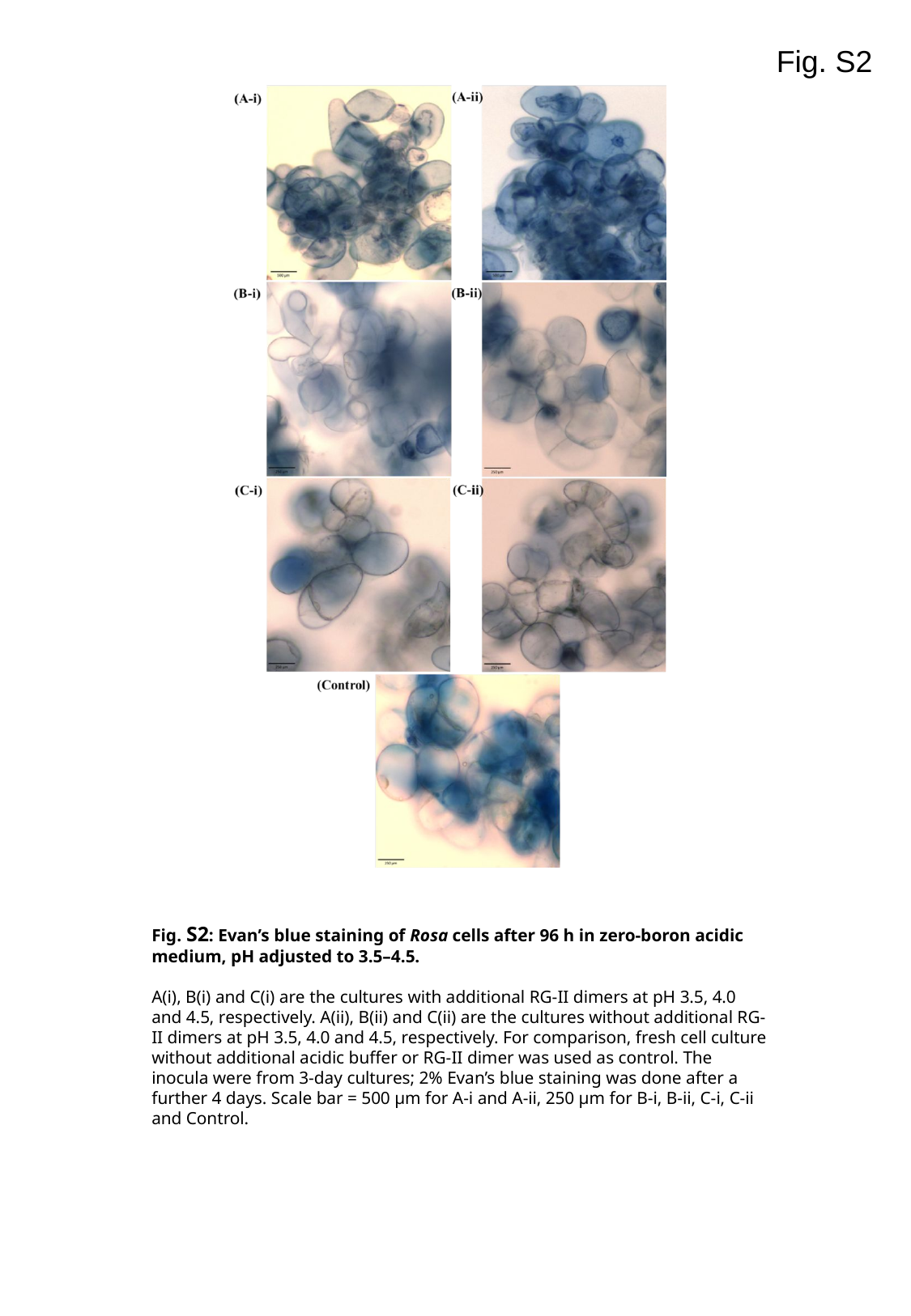

Fig. S2
Fig. S2: Evan’s blue staining of Rosa cells after 96 h in zero-boron acidic medium, pH adjusted to 3.5–4.5.
A(i), B(i) and C(i) are the cultures with additional RG-II dimers at pH 3.5, 4.0 and 4.5, respectively. A(ii), B(ii) and C(ii) are the cultures without additional RG-II dimers at pH 3.5, 4.0 and 4.5, respectively. For comparison, fresh cell culture without additional acidic buffer or RG-II dimer was used as control. The inocula were from 3-day cultures; 2% Evan’s blue staining was done after a further 4 days. Scale bar = 500 µm for A-i and A-ii, 250 µm for B-i, B-ii, C-i, C-ii and Control.

## Slide 3
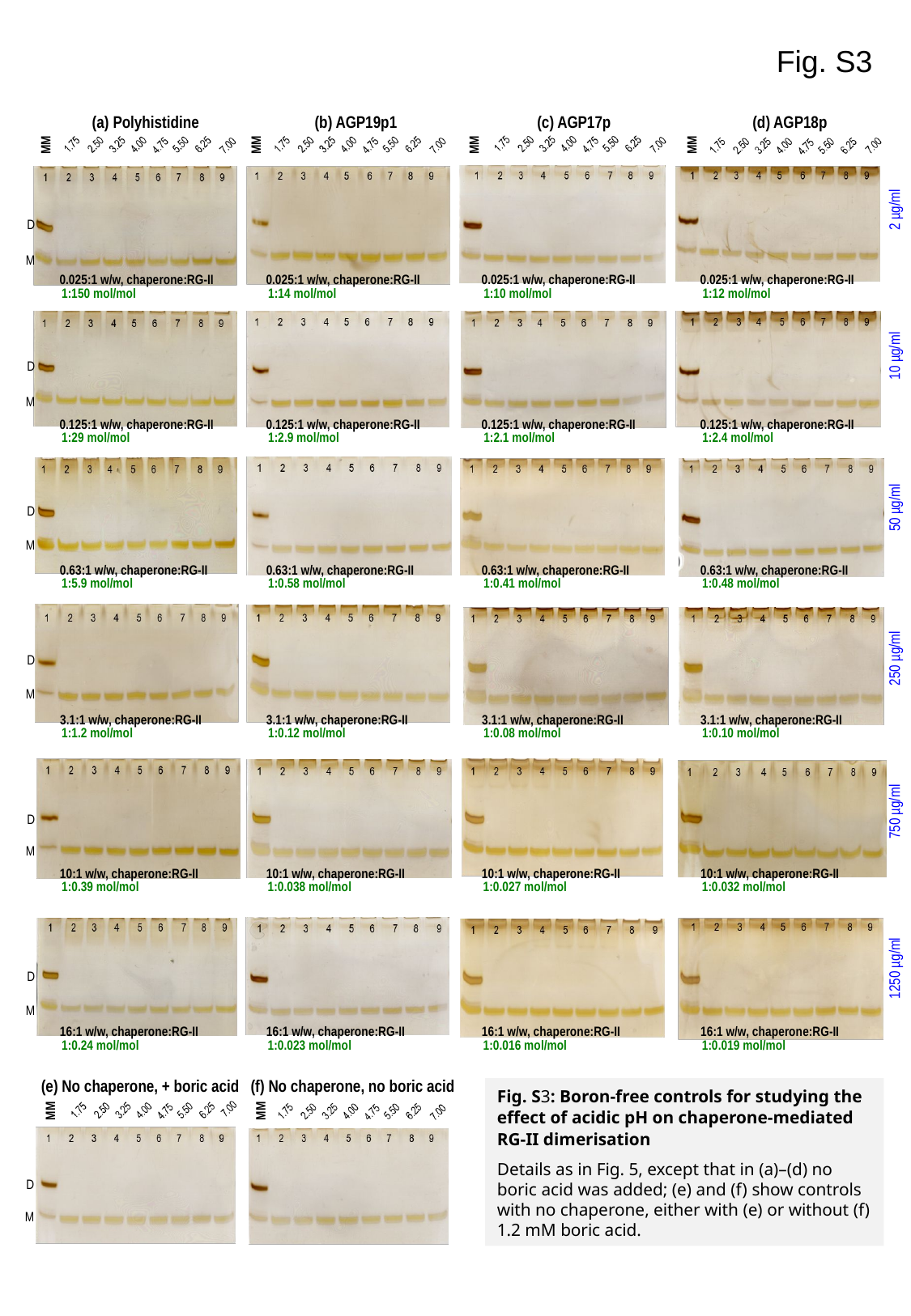

Fig. S3
(a) Polyhistidine
(b) AGP19p1
(c) AGP17p
(d) AGP18p
MM
MM
MM
MM
2 µg/ml
D
M
0.025:1 w/w, chaperone:RG-II
0.025:1 w/w, chaperone:RG-II
0.025:1 w/w, chaperone:RG-II
0.025:1 w/w, chaperone:RG-II
1:150 mol/mol
1:14 mol/mol
1:10 mol/mol
1:12 mol/mol
10 µg/ml
D
M
0.125:1 w/w, chaperone:RG-II
0.125:1 w/w, chaperone:RG-II
0.125:1 w/w, chaperone:RG-II
0.125:1 w/w, chaperone:RG-II
1:29 mol/mol
1:2.9 mol/mol
1:2.1 mol/mol
1:2.4 mol/mol
50 µg/ml
D
M
0.63:1 w/w, chaperone:RG-II
0.63:1 w/w, chaperone:RG-II
0.63:1 w/w, chaperone:RG-II
0.63:1 w/w, chaperone:RG-II
1:5.9 mol/mol
1:0.58 mol/mol
1:0.41 mol/mol
1:0.48 mol/mol
250 µg/ml
D
M
3.1:1 w/w, chaperone:RG-II
3.1:1 w/w, chaperone:RG-II
3.1:1 w/w, chaperone:RG-II
3.1:1 w/w, chaperone:RG-II
1:1.2 mol/mol
1:0.12 mol/mol
1:0.08 mol/mol
1:0.10 mol/mol
750 µg/ml
D
M
10:1 w/w, chaperone:RG-II
10:1 w/w, chaperone:RG-II
10:1 w/w, chaperone:RG-II
10:1 w/w, chaperone:RG-II
1:0.39 mol/mol
1:0.038 mol/mol
1:0.027 mol/mol
1:0.032 mol/mol
1250 µg/ml
D
M
16:1 w/w, chaperone:RG-II
16:1 w/w, chaperone:RG-II
16:1 w/w, chaperone:RG-II
16:1 w/w, chaperone:RG-II
1:0.24 mol/mol
1:0.023 mol/mol
1:0.016 mol/mol
1:0.019 mol/mol
(e) No chaperone, + boric acid
(f) No chaperone, no boric acid
Fig. S3: Boron-free controls for studying the effect of acidic pH on chaperone-mediated RG-II dimerisation
Details as in Fig. 5, except that in (a)–(d) no boric acid was added; (e) and (f) show controls with no chaperone, either with (e) or without (f) 1.2 mM boric acid.
MM
MM
D
M

## Slide 4
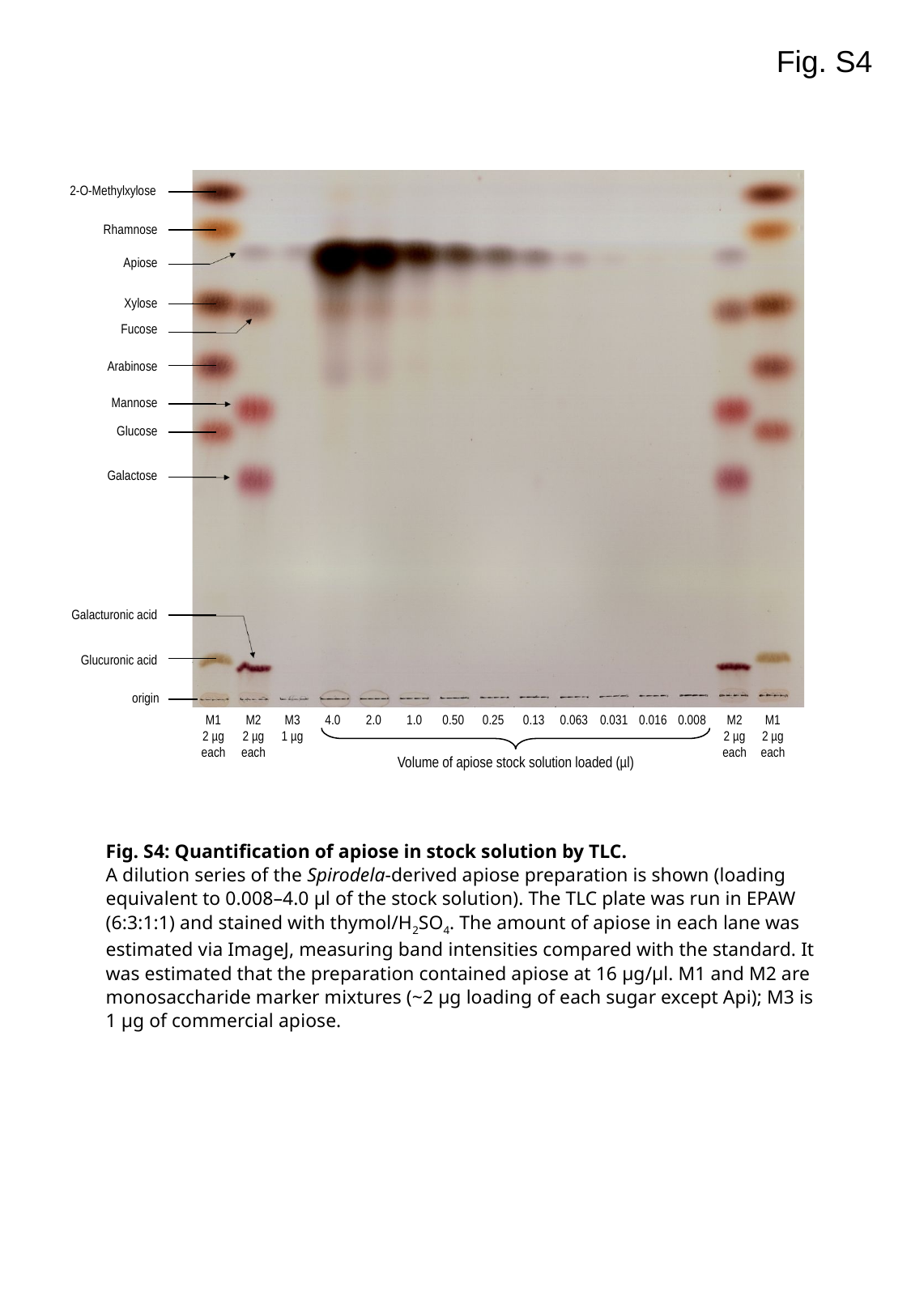

Fig. S4
2-O-Methylxylose
Rhamnose
Apiose
Xylose
Fucose
Arabinose
Mannose
Glucose
Galactose
Galacturonic acid
Glucuronic acid
origin
M1
2 µg
each
M2
2 µg
each
M3
1 µg
4.0
2.0
1.0
0.50
0.25
0.13
0.063
0.031
0.016
0.008
M2
2 µg
each
M1
2 µg
each
Volume of apiose stock solution loaded (µl)
Fig. S4: Quantification of apiose in stock solution by TLC.
A dilution series of the Spirodela-derived apiose preparation is shown (loading equivalent to 0.008–4.0 µl of the stock solution). The TLC plate was run in EPAW (6:3:1:1) and stained with thymol/H2SO4. The amount of apiose in each lane was estimated via ImageJ, measuring band intensities compared with the standard. It was estimated that the preparation contained apiose at 16 µg/µl. M1 and M2 are monosaccharide marker mixtures (~2 µg loading of each sugar except Api); M3 is 1 µg of commercial apiose.

## Slide 5
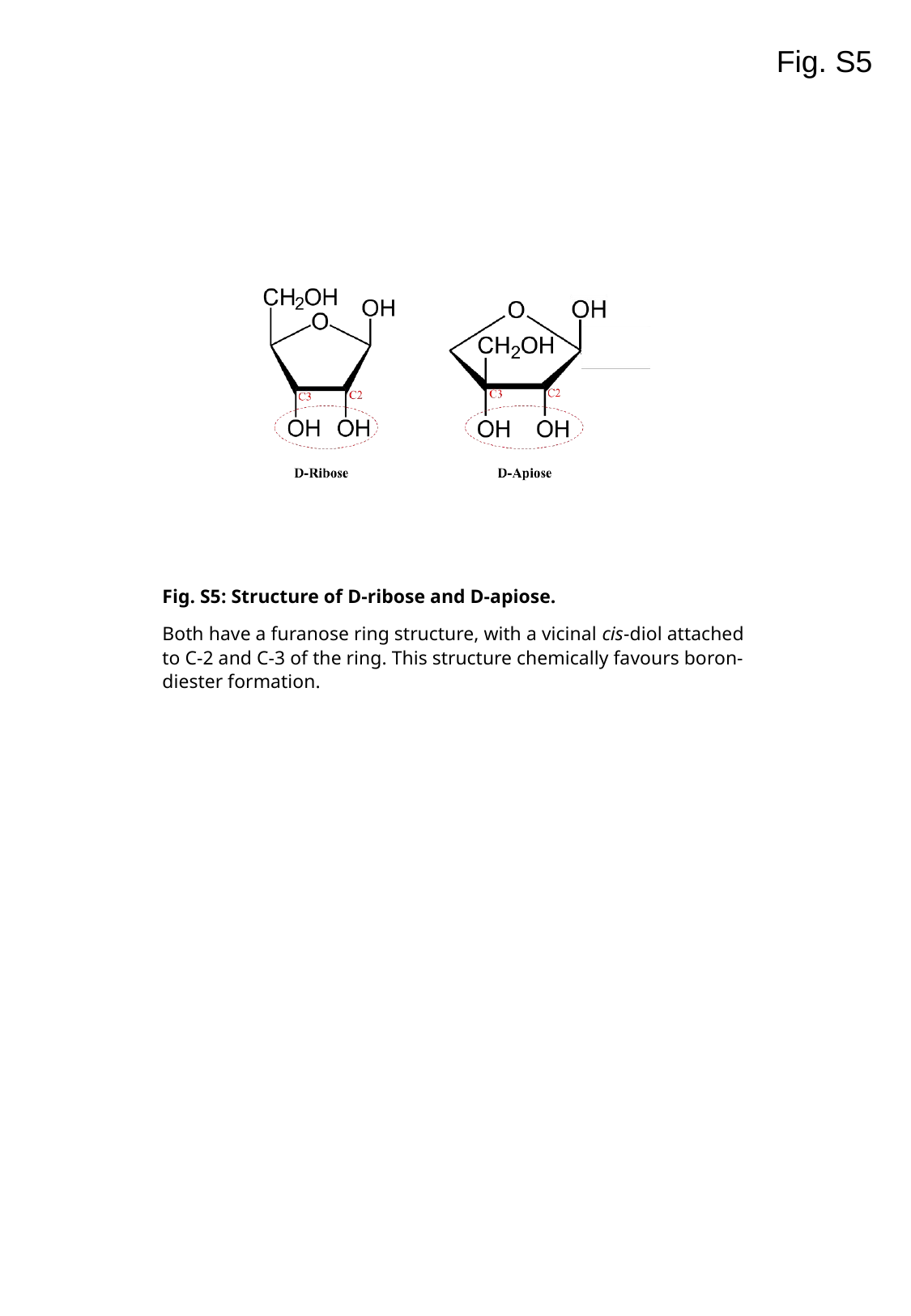

Fig. S5
Fig. S5: Structure of d-ribose and d-apiose.
Both have a furanose ring structure, with a vicinal cis-diol attached to C-2 and C-3 of the ring. This structure chemically favours boron-diester formation.

## Slide 6
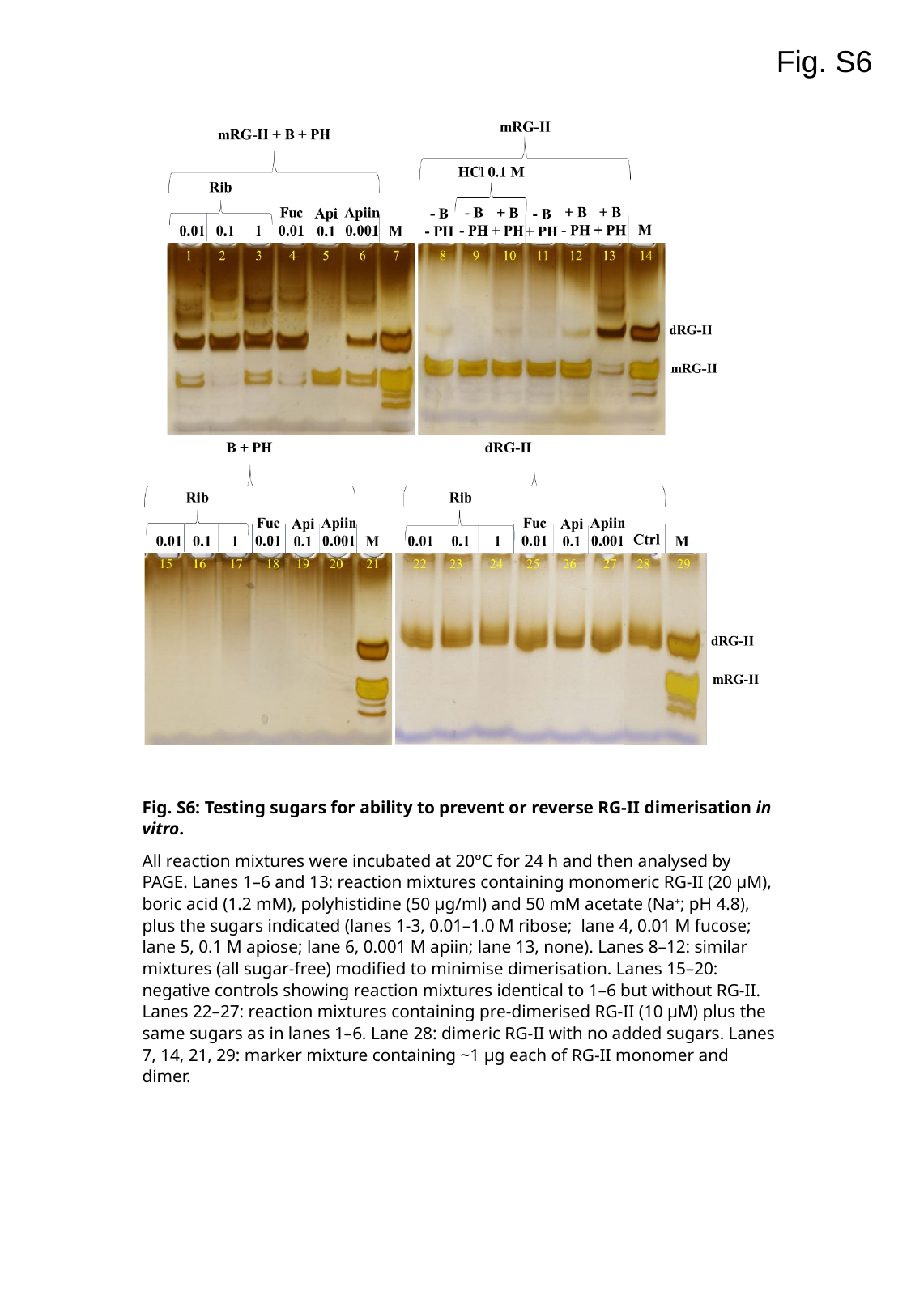

Fig. S6
Fig. S6: Testing sugars for ability to prevent or reverse RG-II dimerisation in vitro.
All reaction mixtures were incubated at 20°C for 24 h and then analysed by PAGE. Lanes 1–6 and 13: reaction mixtures containing monomeric RG-II (20 µM), boric acid (1.2 mM), polyhistidine (50 µg/ml) and 50 mM acetate (Na+; pH 4.8), plus the sugars indicated (lanes 1-3, 0.01–1.0 M ribose; lane 4, 0.01 M fucose; lane 5, 0.1 M apiose; lane 6, 0.001 M apiin; lane 13, none). Lanes 8–12: similar mixtures (all sugar-free) modified to minimise dimerisation. Lanes 15–20: negative controls showing reaction mixtures identical to 1–6 but without RG-II. Lanes 22–27: reaction mixtures containing pre-dimerised RG-II (10 µM) plus the same sugars as in lanes 1–6. Lane 28: dimeric RG-II with no added sugars. Lanes 7, 14, 21, 29: marker mixture containing ~1 µg each of RG-II monomer and dimer.

## Slide 7
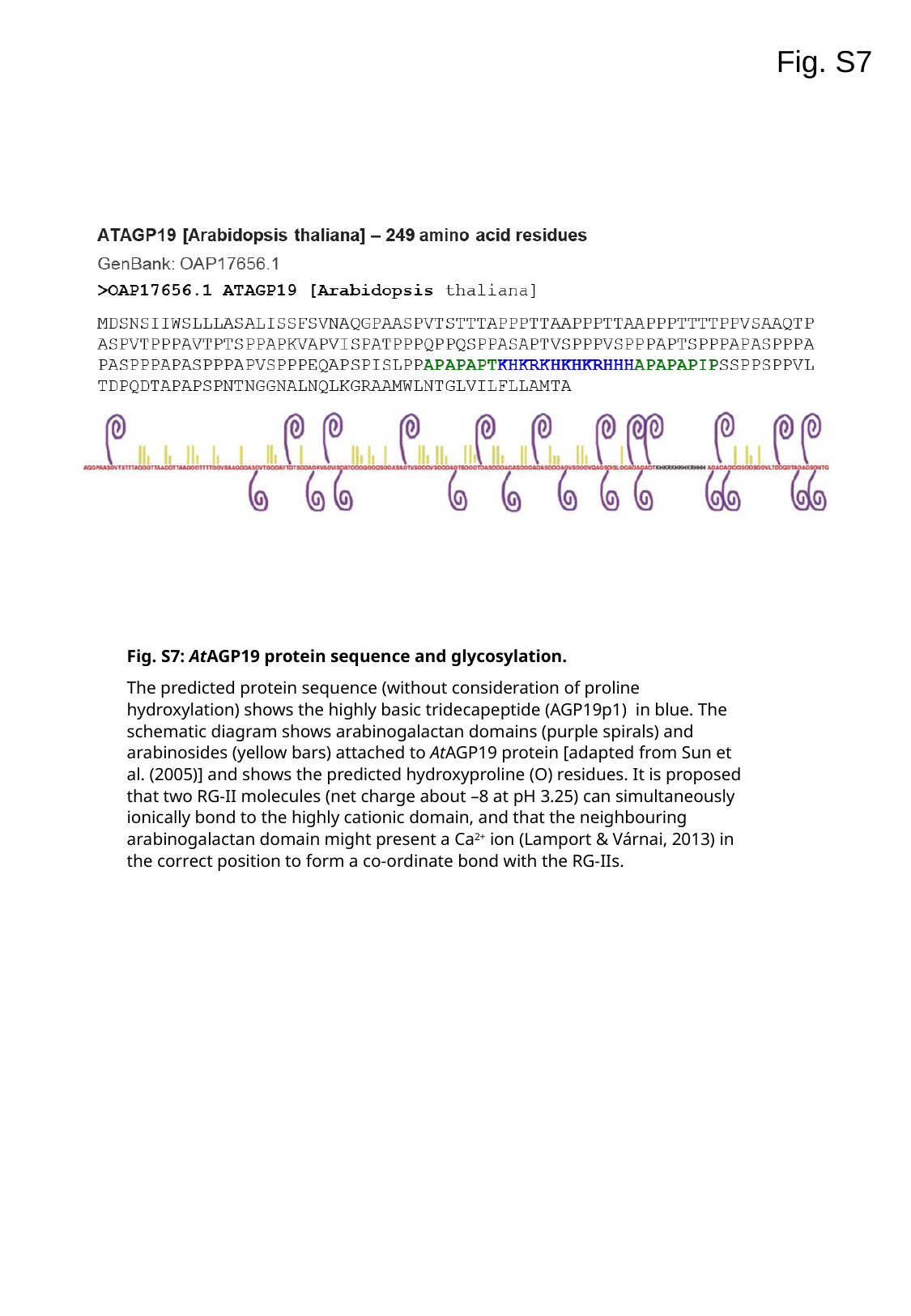

Fig. S7
Fig. S7: AtAGP19 protein sequence and glycosylation.
The predicted protein sequence (without consideration of proline hydroxylation) shows the highly basic tridecapeptide (AGP19p1) in blue. The schematic diagram shows arabinogalactan domains (purple spirals) and arabinosides (yellow bars) attached to AtAGP19 protein [adapted from Sun et al. (2005)] and shows the predicted hydroxyproline (O) residues. It is proposed that two RG-II molecules (net charge about –8 at pH 3.25) can simultaneously ionically bond to the highly cationic domain, and that the neighbouring arabinogalactan domain might present a Ca2+ ion (Lamport & Várnai, 2013) in the correct position to form a co-ordinate bond with the RG-IIs.
