# Supplementary material for: Making and breaking of boron bridges in the pectic domain rhamnogalacturonan‐II at apoplastic pH in vivo and in vitro
Source: Plant J. 2023 Feb 8;113(6):1310–29. doi: 10.1111/tpj.16112 (PMC10952590; doi:10.1111/tpj.16112)
Supplement: Supplementary file 2 — Table S1. Effect of pH on solubility of Pb2+ with and without boric acid. Table S2. Typical composition of in‐vitro reaction mixtures with monomeric RG‐II, boric acid, and cationic chaperones to study dimerization. [file TPJ-113-1310-s001.docx]

**Begum *et al.*, Supplementary Tables**

Table S1. Effect of pH on solubility of lead [initially as Pb(NO_3_)_2_] with and without boric acid*

|  | Pb(NO_3_)_2_ | Precipitate visible after 24 h at pH..... | | | | | | | | | |
| --- | --- | --- | --- | --- | --- | --- | --- | --- | --- | --- | --- |
|  | (µM) | 1.7 | 2.3 | 2.9 | 3.2 | 3.9 | 4.6 | 5.3 | 6.1 | 6.8 | 7.0 |
|  |  |  |  |  |  |  |  |  |  |  |  |
| no boric acid | 0 | – | – | – | – | – | – | – | – | – | – |
|  | 100 | – | – | – | – | – | – | – | – | – | – |
|  | 500 | – | – | – | – | – | – | – | – | – | – |
|  | 2500 | – | – | – | – | – | – | – | – | – | +++ |
|  |  |  |  |  |  |  |  |  |  |  |  |
| with 1.2 mM boric acid | 0 | – | – | – | – | – | – | – | – | – | – |
|  | 100 | – | – | – | – | – | – | – | – | – | – |
|  | 500 | – | – | – | – | – | – | – | – | – | – |
|  | 2500 | – | – | – | – | – | – | – | – | + | +++ |

*Lead nitrate (with or without 1.2 mM boric acid) was dissolved in buffers similar to those mentioned in Fig. 3 and incubated overnight at 20°C. The mixtures were then checked for visible precipitates. The solubility of Pb(OH)_2_ is reported as 640 µM (Handbook of Chemistry and Physics, 1st edition, 2000, CRC Press ISBN 0-8493-0740-6)

Table S2. Typical composition of *in-vitro* reaction mixtures with monomeric RG-II, boric acid and cationic chaperones to study dimerisation

| RG-II monomer | Peptide concen­tration | *Corresponding peptide molarity* | | | | Other components typically used | | | |
| --- | --- | --- | --- | --- | --- | --- | --- | --- | --- |
|  |  | *PolyHis* | *AGP19p1* | *AGP17p* | *AGP18p* |  | Boric acid | Pb(NO_3_)_2_ | buffer* |
| (M_r_ 5000) |  | *(M_r_ 18400)* | *(M_r_ 1790)* | *(M_r_ 1290)* | *(M_r_ 1480)* |  | (M_r_ 68) | (M_r_ 331) |  |
| µM* | µg/ml | *µM* | *µM* | *µM* | *µM* |  | µM* | µM* | mM |
| 16 | 2 | *0.109* | *1.12* | *1.55* | *1.35* |  | 1200 | 500 | 50 |
| 16 | 10 | *0.543* | *5.59* | *7.75* | *6.76* |  | 1200 | 500 | 50 |
| 16 | 50 | *2.72* | *27.9* | *38.8* | *33.8* |  | 1200 | 500 | 50 |
| 16 | 250 | *13.6* | *140* | *194* | *169* |  | 1200 | 500 | 50 |
| 16 | 750 | *40.8* | *419* | *581* | *507* |  | 1200 | 500 | 50 |
| 16 | 1250 | *67.9* | *698* | *969* | *845* |  | 1200 | 500 | 50 |

Yellow = maximum dimerisation at optimal pH.

*Varies; see individual experiments.
